# Icariside II Ameliorates Cognitive Impairments Induced by Chronic Cerebral Hypoperfusion by Inhibiting the Amyloidogenic Pathway: Involvement of BDNF/TrkB/CREB Signaling and Up-Regulation of PPARα and PPARγ in Rats

**DOI:** 10.3389/fphar.2018.01211

**Published:** 2018-10-23

**Authors:** Caixia Yin, Yuanyuan Deng, Yuangui Liu, Jianmei Gao, Lingli Yan, Qihai Gong

**Affiliations:** ^1^Key Laboratory of Basic Pharmacology of Ministry of Education and Joint International Research Laboratory of Ethnomedicine of Ministry of Education, Zunyi Medical University, Zunyi, China; ^2^School of Pharmacy, Zunyi Medical University, Zunyi, China

**Keywords:** icariside II, chronic cerebral hypoperfusion, bilateral common carotid artery occlusion, beta-amyloid, peroxisome proliferator-activated receptor

## Abstract

Chronic cerebral hypoperfusion (CCH) is regarded as a high-risk factor for cognitive decline of vascular dementia (VD) as it is conducive to induce beta-amyloid (Aβ) aggregation. Icariside II (ICS II), a plant-derived flavonoid compound, has showed neuroprotective effect on animal models of Alzheimer’s disease (AD) by decreasing Aβ levels. Here, we assessed the effect of ICS II on CCH-induced cognitive deficits and Aβ levels in rats, and the possible underlying mechanisms were also explored. It was disclosed that CCH induced by bilateral common carotid artery occlusion (BCCAO) caused cognitive deficits, neuronal injury and increase of Aβ_1-40_ and Aβ_1-42_ levels in the rat hippocampus, while oral administration of ICS II for 28 days abolished the above deficits in the hippocampus of BCCAO rats. Meanwhile, ICS II significantly decreased the expression of beta-amyloid precursor protein (APP) and β-site amyloid precursor protein cleavage enzyme 1 (BACE1), as well as increased the expression of a disintegrin and metalloproteinase domain 10 (ADAM10) and insulin-degrading enzyme (IDE). ICS II also activated peroxisome proliferator-activated receptor (PPAR)α and PPARγ, enhanced the expression of brain-derived neurotrophic factor (BDNF), tyrosine receptor kinase B (TrkB), levels of Akt and cAMP response element binding protein (CREB) phosphorylation. Together, these findings suggested that ICS II attenuates CCH-induced cognitive deficits by inhibiting the amyloidogenic pathway via involvement of BDNF/TrkB/CREB signaling and up-regulation of PPARα and PPARγ in rats.

## Introduction

Vascular dementia (VD), a degenerative cerebrovascular disorder caused by hypoperfusive, ischemic, or hemorrhagic brain injury, is characterized by memory decline and cognitive dysfunction (Kaur et al., 2016). VD is the second most leading cause of dementia following Alzheimer’s disease (AD), accounting for 20–30% of total dementia cases among the elderly population, which has become a huge economic burden to the families and the society (Saxena et al., 2015; Wu et al., 2016; Hu et al., 2017). Although the etiopathogenetic mechanism of VD is complicated and remains elusive, growing studies have shown that the pathological process of VD is associated with chronic cerebral hypoperfusion (CCH) resulting from a chronic state of cerebral blood flow reduction which is found in patients with mild cognitive impairment (Staffen et al., 2006; Zhao and Gong, 2015). Moreover, several evidences suggested that CCH could induce progressive cognitive decline accompanied by beta-amyloid (Aβ) aggregation (Ai et al., 2013). Notably, bilateral common carotid artery occlusion (BCCAO) can successfully imitate CCH in rats, which is generally taken as a suitable model of VD with significant reduction of cerebral blood flow and characteristic neuronal damage in the hippocampus (Saxena et al., 2015; Mohamed et al., 2018). Although existing therapeutic agents such as memantine, galantamine, donepezil, and rivastigmine have achieved significant therapeutic advances in VD, they are limited in safety and effectiveness of clinical application (Baskys and Cheng, 2012). Thus, it is urgent to explore new agents with improved efficacy and safety profiles for the treatment of VD.

*Herba Epimedii* (family, Berberidaceae; common name, Yinyanghuo in China) is a well-known traditional Chinese medicine in the treatment of hypertension, osteoporosis, sexual dysfunction and dementia (Chen et al., 2016; Deng et al., 2017). Icariside II (ICS II), a prenylated flavonoid glycoside extracted from *Herba Epimedii*, has been demonstrated to exhibit a broad-spectrum pharmacological activities including anti-cancer, anti-inflammatory, anti-hypoxia, and anti-osteoporosis (Luo et al., 2016). Interestingly, our previous studies have shown that ICS II improves cognitive deficits, attenuates neuronal death, and decreases the levels of Aβ_1-40_ and Aβ_1-42_ in rat hippocampus subjected to intracerebroventricular streptozotocin (Yin et al., 2016), and ICS II pretreatment also protects against middle cerebral artery occlusion (MCAO)-induced cerebral ischemic/reperfusion injury *via* up-regulation of peroxisome proliferator-activated receptor (PPAR) α and PPARγ in rats (Deng et al., 2016). However, whether ICS II is effective in improving VD-associated cognitive deficits remains unknown.

This study was designed to evaluate whether ICS II has therapeutic effect on VD-associated cognitive deficits induced by CCH and further to explore its possible molecular mechanisms.

## Materials and Methods

### Reagents

ICS II (purity ≥98% *via* HPLC) was provided from Nanjing Zelang Medical Technology Corporation Ltd. (Nanjing, China) and dissolved in physiological saline with 30-min ultra-sonication. All other experimental reagents were of analytical grade and commercially available.

### Animals

Healthy male Sprague-Dawley (SD) rats (age, 3 months old; weight, 250 ± 20 g) were purchased from the Experimental Animal Center of the Third Military Medical University (Chongqing, China; Certificate No. SCXK2012-0011). Rats were kept five per cage in a room of SPF- grade, and the room temperature was maintained at 23 ± 1°C under a 12 h light/dark cycle. Rats were allowed free access to standard rodent chow and water. All animal experiments were performed in accordance with the State Committee of Science and Technology of the People’s Republic of China Order No. 2 on November 14, 1988 (revised in 2011), and the protocol was approved by the Experimental Animal Ethics Committee of the Zunyi Medical University. All efforts were made to minimize animal pain and animal use.

### Surgery

After rats were anesthetized with sodium pentobarbital (50 mg/kg, i.p.), BCCAO (a 2-vessel occlusion model of global cerebral ischaemia) surgery was carried out as previously described (Ashok et al., 2016; Luo et al., 2016; Akinmoladun et al., 2018). Briefly, the rats were placed in a supine position, both right and left common carotid arteries were exposed between the sternomastoid and sternohyoid muscles *via* a 2 cm midline neck incision, and carefully separated from vagal nerves, carotid sheath and cervical sympathetic. A small-diameter silk suture was passed below each carotid artery and was used to irreversibly ligate the arteries to induce ischaemia. The sham-operated rats were treated similarly to the ischemia rats without the ligation of two carotid arteries. The body temperature of rats was maintained at 37 ± 0.5°C during the surgery using a warm pad.

### Drug Administration and Experimental Design

The rats were randomly assigned to the following six groups: sham group (*n* = 13), sham + ICS II 16 group (*n* = 13), BCCAO group (*n* = 13), BCCAO + ICS II 4 group (*n* = 13), BCCAO + ICS II 8 group (*n* = 13), and BCCAO + ICS II 16 group (*n* = 13). The CCH model was developed on the 10th day after BCCAO. Thereafter, rats in BCCAO + ICS II 4 group, BCCAO + ICS II 8 group, and BCCAO + ICS II 16 group were intragastrically administered with ICS II at doses of 4, 8, and 16 mg/kg daily for 28 consecutive days. sham + ICS II 16 group received the same treatment as BCCAO + ICS II 16 group, while sham and BCCAO groups were administered with volume-matched physiological saline, instead. In addition, as a supplemental experiment, rats were randomly divided into three groups: sham group (*n* = 7), BCCAO group (*n* = 7), and BCCAO + ICS II 16 group (*n* = 7). Rats in BCCAO + ICS II 16 group were intragastrically administered with ICS II daily at the dose of 16 mg/kg on the 10th day after BCCAO for 28 consecutive days, while rats in sham and BCCAO groups were sacrificed on the 10th day after BCCAO.

### Morris Water Maze Test

The Morris water maze was used for the assessment of spatial learning and memory capability as previously described (Yan et al., 2017). The task was performed during days 33–37 after BCCAO. The water maze test was carried out in a circular black pool (160 cm in diameter and 50 cm in height), which was partially filled with water (24 ± 2°C). The pool was divided into four equal quadrants, and a hidden platform (12 cm in diameter) was placed at 1 cm under the surface of the water in the midpoint of the target quadrant. The procedure of Morris water maze test included two steps. The place navigation test was conducted three times daily for four consecutive days known as the first step, and the escape latency (time of reaching the hidden platform) and the swimming speed of rats were recorded. Rats were expected to find the hidden platform during a maximum period of 120 s and were allowed to rest on the platform for 15 s during the place navigation test. If a rat did not succeed in finding the platform within 120 s, its escape latency was recorded as 120 s, and it was then gently placed on the platform by hand for guidance. The spatial probe test was performed on the 5th day with the removal of the platform known as the second step, and the swimming distances, the time spent in the target quadrant and the swimming speed of rats were recorded by the TopScan-Topview Behavior Analyzing System (TopScan Version 3.00).

### Morphometric Analysis

Following the Morris water maze test, rats (*n* = 4 in each group) were anesthetized using sodium pentobarbital and transcardially perfused in 0.1 M phosphate-buffered saline followed by 4% paraformaldehyde (pH 7.4). The brains were immediately taken out from the skull and immersed in 4% paraformaldehyde for 1 week. Then, the brains were fixed using paraffin, which were sectioned into 3 μm slices along coronal plane. The coronal sections were used for HE staining (Li W. X. et al., 2015) and Nissl staining (Deng et al., 2017), respectively. A light microscope (KS300, Zeiss-Kontron, Germany) was used to examine the histopathological abnormalities of neurons in the hippocampus.

### Western blot

After rats were sacrificed, the hippocampus tissues were dissected and collected at -80°C, which were used for Western blot analysis as described previously (Li F. et al., 2015). The hippocampus tissues were lysed in the RIPA lysis buffer (pH = 8, 50mM Tris-HCl, 150 mM NaCl, 0.5% deoxycholate, 0.1% SDS, 1% Triton X-100, protease inhibitor cocktail), which is widely used to extract soluble proteins. BCA protein assay kit was utilized to analyze the protein concentrations. 30 μg protein of each sample was heat-blocked in a loading buffer at 95°C for 5 min, and separated by 8–12% sodium dodecyl sulfate-polyacrylamide gel electrophoresis. Then, the sample was transferred into a PVDF membrane, which was blocked for 2 h in 5% non-fat milk. Immunoblots were probed using primary antibodies against: Aβ_1-42_ (1:1000, Abcam, United States), Aβ_1-40_ (1:1000, Abcam, United States), IDE (1:1000, Abcam, United States), BACE1 (1:1000, Abcam, United States), ADAM10 (1:1000, Abcam, United States), APP (1:1000, Sangon Biotech, China), PPARα (1:1000, Abcam, United States), PPARβ (1:1000, Abcam, United States), PPARγ (1:1000, Abcam, United States), BDNF (1:500, Abcam, United States), TrkB (1:500, Abcam, United States), Akt (1:1000, Sangon Biotech, China), p-Akt (1:1000, Sangon Biotech, China), CREB (1:500, Cell Signaling Technology, United States), p-CREB (1:500, Cell Signaling Technology, United States), β-actin (1:5000, Beyotime, China), and GAPDH (1:5000, Beyotime, China). Subsequently, the membranes were washed with TBST buffer and then incubated in HRP-conjugated anti-rabbit secondary antibody (1:2000, Beyotime, China) or anti-mouse secondary antibody (1:5000, Beyotime, China) for 1 h at room temperature. Finally, the immunoreactive bands were exposed using the enhanced chemiluminescence reagents (ECL, Beyotime, China) and digitized using Quantity One-4.6.7. (Bio-Rad, United States).

### Statistical Analysis

Statistical analyses were carried out using SPSS 16.0 analysis software and all results were presented as mean ± SEM. The escape latency of MWM data were analyzed using a repeated measures analysis of variance (ANOVA) process. One-way ANOVA was used for the analysis of other data, and *post hoc* least significant difference (LSD) analysis was used to compare differences between groups. The significant differences were set at *P* < 0.05.

## Results

### ICS II Alleviated BCCAO-Induced Spatial Learning and Memory Impairments

**FIGURE 1:**
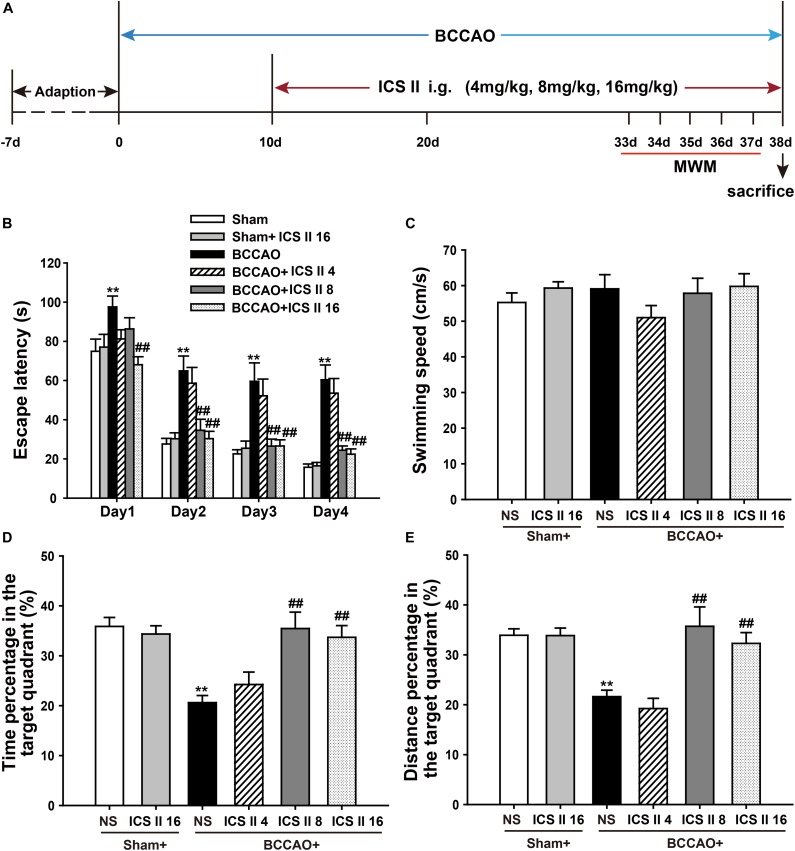
Icariside (ICS) II alleviated BCCAO-induced spatial learning and memory impairments. **(A)** Experimental design: Rats received BCCAO surgery and then were given 4, 8, and 16 mg/kg ICS II daily for 28 days. Morris water maze was carried out for five consecutive days from day 33 after BCCAO surgery. **(B)** The escape latency of the rats to reach the hidden platform from days 1 to 4. **(C)** The average swimming speed. **(D,E)** The percentages of time and distances in the target quadrant. Data were expressed as mean ± SEM (*n* = 13). ^∗∗^*P* < 0.01 vs. sham, ^##^*P* < 0.01 vs. BCCAO.

To investigate whether ICS II could alleviate spatial learning and memory impairments induced by BCCAO, a 5-day MWM test was performed from day 33 after surgery (Figure 1). In the place navigation test, the *P*-value of Mauchly’s Test of Sphericity for escape latency was 0.025 < 0.05, which accepted Greenhouse–Geisser correction. Tests of Within-Subjects Effects in the “days factor” and the interaction of “days × groups” showed significant differences with corresponding values of [*F*(2.668,192.090) = 136.299, *P* < 0.001] and [*F*(13.340,192.090) = 1.779, *P* = 0.047], respectively. Tests of Between-Subjects Effects in the “latency between groups” showed a significant difference [*F*(5,72) = 15.272, *P* < 0.001]. As shown in Figure 1B, the escape latency of rats from sham and sham + ICS II 16 groups had not significant differences, while BCCAO rats showed a longer escape latency as compared to the sham group from days 1 to 4 (*P* < 0.01; *P* < 0.01; *P* < 0.01; *P* < 0.01). However, ICS II treatment abolished the escape latency increase on days 1, 2, 3, and 4, while ICS II 4 mg/kg in BCCAO rats had no effect.

In the spatial probe test, the swimming speeds from these six groups did not show differences [*F*(5,72) = 0.935, *P* = 0.463] (Figure 1C). The percentages of time spent in target quadrant and target quadrant distance on the 5th day were markedly reduced after BCCAO, which were significantly prevented by ICS II at the dosage of 8 and 16 mg/kg [*F*(5,72) = 7.828, *P* < 0.001; *F*(5,72) = 9.462, *P* < 0.001, respectively] (Figures 1D,E). Taken together, these results indicated that spatial learning and memory impairments were apparent in BCCAO rats, which were dose-dependently prevented by ICS II treatment.

### ICS II Attenuated Neuronal Death in the Hippocampus in BCCAO-Treated Rats

**FIGURE 2:**
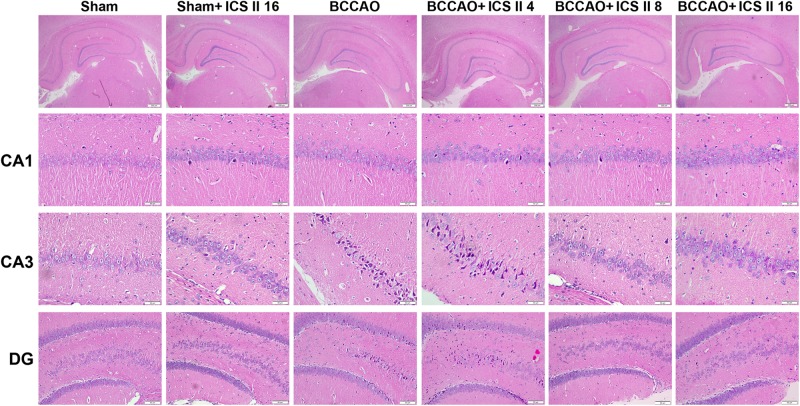
Icariside II attenuated BCCAO-induced morphological alterations in the hippocampus. The sections of hippocampus CA1, CA3, and DG region were obtained and stained by HE (magnification 40× or 400×, scale bar = 200 μm or 50 μm).

**FIGURE 3:**
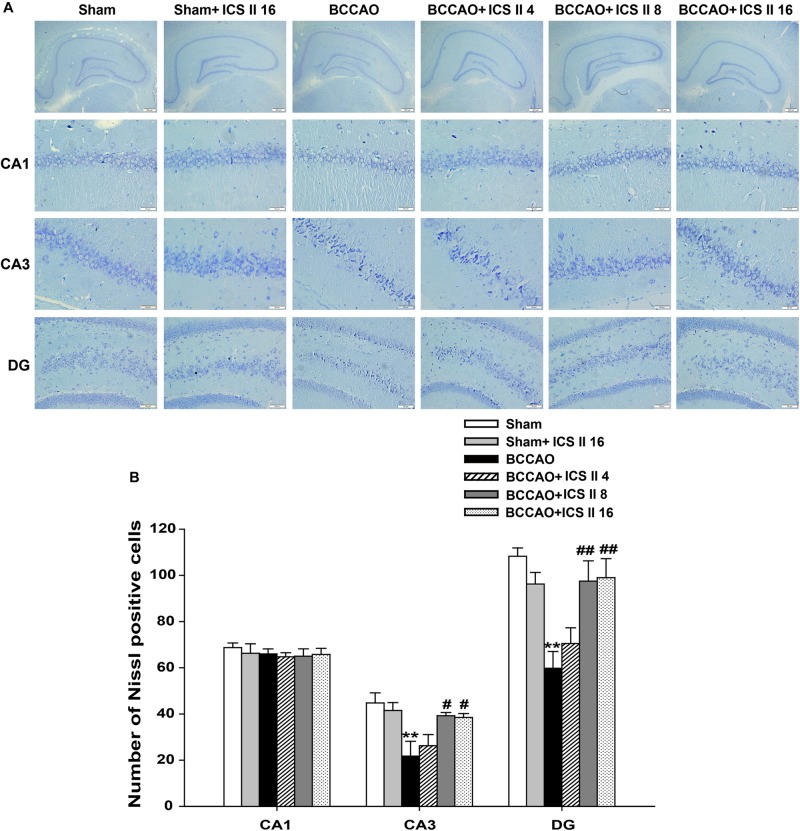
Icariside II attenuated BCCAO-induced neuronal death in the hippocampus. Nissl staining was used to examine the neuronal death in hippocampal CA1, CA3, and DG regions. **(A)** The sections of hippocampal CA1, CA3, and DG regions (magnification 40× or 400×, scale bar = 200 μm or 50 μm). **(B)** Quantitative analysis of Nissl bodies in hippocampal CA1, CA3, and DG regions. Data were expressed as mean ± SEM (*n* = 4). ^∗∗^*P* < 0.01 vs. sham, ^#^*P* < 0.05, ^##^*P* < 0.01 vs. BCCAO.

Bilateral common carotid artery occlusion-induced neuronal death in the hippocampus was examined by HE and Nissl staining. HE staining indicated normal morphology and clear boundary of the neurons in CA3 and DG regions of the sham and sham + ICS II 16 groups, while the BCCAO rats exhibited abnormal neuronal morphology with shrunken neurons, nuclear dark staining and even disappearance. Interestingly, treatment with ICS II 8 and 16mg/kg partially prevented such neuronal changes (Figure 2). Nissl staining revealed that the hippocampal neurons in CA3 and DG regions had intact cellular structures and abundant Nissl bodies in sham rats. In contrast, the Nissl bodies in CA3 and DG regions were reduced and neurons appeared atrophic and loosely arranged in BCCAO rats [*F*(5,81) = 6.266, *P* < 0.001; *F*(5,81) = 8.323, *P* < 0.001, respectively] (*P* < 0.01; *P* < 0.01). Treatment with ICS II 8 and 16 mg/kg increased the number of the Nissl bodies in CA3 and DG regions compared with the BCCAO group (Figures 3A,B) (*P* < 0.05; *P* < 0.05; *P* < 0.01; *P* < 0.01). These findings suggested that ICS II at doses of 8 and 16 mg/kg could attenuate BCCAO-induced neuronal death in CA3 and DG regions of hippocampus.

### ICS II Prevented BCCAO-Induced the Levels of Aβ Oligomers in the Hippocampus

**FIGURE 4:**
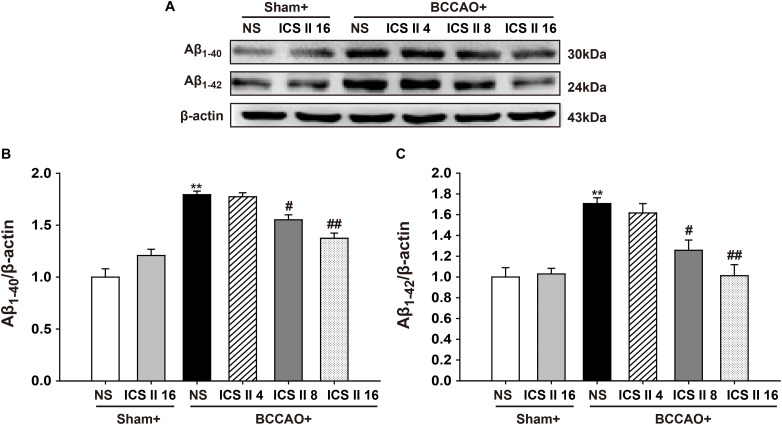
Icariside II prevented the levels of Aβ_1-40_ and Aβ_1-42_ oligomers induced by BCCAO in the hippocampus. The levels of Aβ_1-40_ and Aβ_1-42_ oligomers were examined by Western blot. **(A)** The antibody-reactive band of the Aβ_1-40_ and Aβ_1-42_ oligomers. **(B)** Quantitative analysis of Aβ_1-40_ oligomer levels. **(C)** Quantitative analysis of Aβ_1-42_ oligomer levels. The relative optical density was normalized to β-actin. Data were expressed as mean ± SEM (*n* = 3). ^∗∗^*P* < 0.01 vs. sham, ^#^*P* < 0.05, ^##^*P* < 0.01 vs. BCCAO.

Excessively increased Aβ production is a crucial factor in the pathological process of VD induced by CCH (Li W. X. et al., 2015), as soluble oligomers of Aβ are neurotoxic and proinflammatory, here we examined the contents of Aβ_1-40_ and Aβ_1-42_ oligomers using Western blot. The levels of Aβ_1-40_ and Aβ_1-42_ oligomers in the BCCAO group were higher than those in sham rats [*F*(5,12) = 23.110, *P* < 0.001; *F*(5,12) = 9.409, *P* = 0.001, respectively] (*P* < 0.01; *P* < 0.01). However, ICS II at the doses of 8 and 16 mg/kg markedly prevented the abnormal increase in the levels of Aβ_1-40_ and Aβ_1-42_ induced by BCCAO (*P* < 0.05; *P* < 0.01; *P* < 0.05; *P* < 0.01) (Figures 4A–C).

### ICS II Decreased APP and BACE1 Expression, Increased ADAM10 and IDE Expression in BCCAO-Treated Rats

**FIGURE 5:**
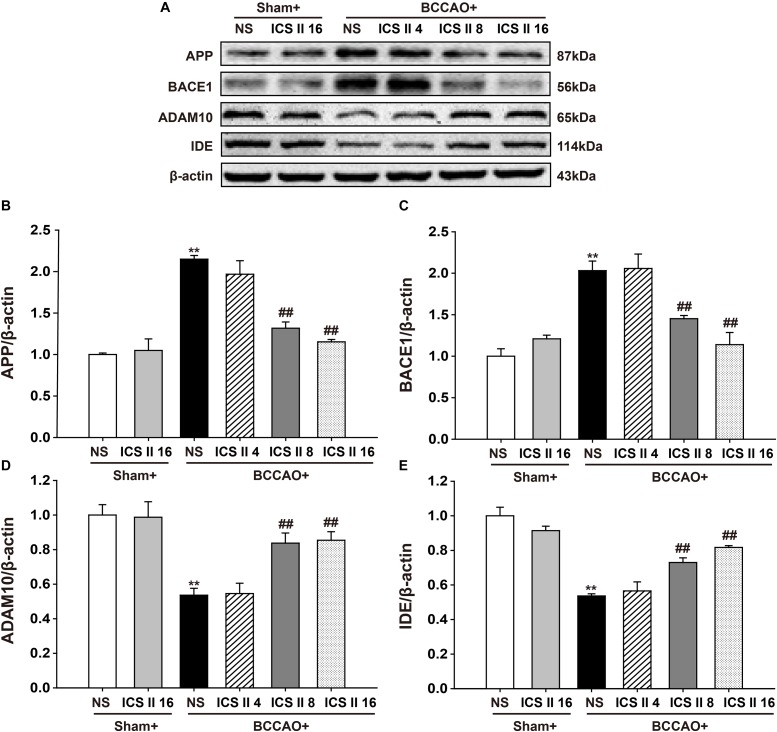
Icariside II decreased APP and BACE1 expression, increased ADAM10 and IDE expression in BCCAO-treated rats. The expression of APP, BACE1, ADAM10, and IDE protein were examined by Western blot. **(A)** The antibody-reactive band of the APP, BACE1, ADAM10, and IDE. **(B)** Quantitative analysis of APP levels. **(C)** Quantitative analysis of BACE1 levels. **(D)** Quantitative analysis of ADAM10 levels. **(E)** Quantitative analysis of IDE levels. The relative optical density was normalized to β-actin. Data were expressed as mean ± SEM (*n* = 3). ^∗∗^*P* < 0.01 vs. sham, ^##^*P* < 0.01 vs. BCCAO.

To understand the mechanisms that ICS II suppressed BCCAO-induced increase of Aβ levels, the protein expressions of APP, BACE1, ADAM10 and IDE were investigated by Western blot. The protein expressions levels of APP and BACE1 were significantly increased by BCCAO [*F*(5,12) = 27.065, *P* < 0.001; *F*(5,12) = 16.562, *P* < 0.001, respectively] (*P* < 0.01; *P* < 0.01), and ICS II at doses of 8 and 16 mg/kg markedly down-regulated them (*P* < 0.01; *P* < 0.01; *P* < 0.01; *P* < 0.01) (Figures 5A–C). In addition, the protein expression levels of ADAM10 and IDE in BCCAO rats were lower than those in the sham rats [*F*(5,12) = 11.347, *P* < 0.001; *F*(5,12) = 20.449, *P* < 0.001, respectively] (*P* < 0.01; *P* < 0.01), and which were up-regulated by ICS II at the dosage of 8 and 16 mg/kg 28 days later (*P* < 0.01; *P* < 0.01; *P* < 0.01; *P* < 0.01) (Figures 5A,D,E). These results suggested that ICS II could reduce Aβ production via the down-regulation of APP and BACE1 and the up-regulation of ADAM10, and it might also increase Aβ degradation via the up-regulation of IDE after BCCAO.

### ICS II Enhanced BDNF/TrkB/CREB Signaling in BCCAO-Treated Rats

**FIGURE 6:**
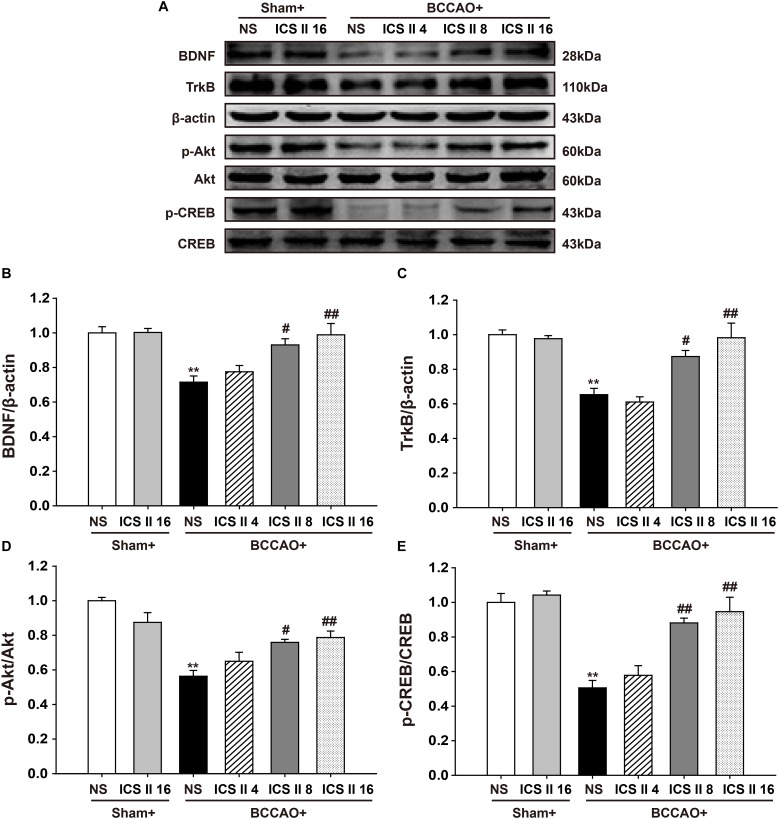
Icariside II enhanced BDNF/TrkB/CREB signaling in BCCAO-treated rats. The expression of BDNF and TrkB protein, as well as the levels of Akt and CREB phosphorylation were examined by Western blot. **(A)** The antibody-reactive band of BDNF and TrkB protein together with Akt and CREB phosphorylation. **(B)** Quantitative analysis of BDNF levels. **(C)** Quantitative analysis of TrkB levels. **(D)** Quantitative analysis of Akt phosphorylation. **(E)** Quantitative analysis of CREB phosphorylation. The relative optical density was normalized to β-actin or Akt or CREB. Data were expressed as mean ± SEM (*n* = 3). ^∗∗^*P* < 0.01 vs. sham, ^#^*P* < 0.05, ^##^*P* < 0.01 vs. BCCAO.

In the brain, the enhancement of BDNF/TrkB/CREB signaling is an important factor in increasing neuroplasticity and promoting long term memory. Here we examined the expression of BDNF and TrkB, as well as levels of Akt and CREB phosphorylation. A significant reduction in BDNF and TrkB levels from BCCAO-induced rats were found compared with those of the sham rats [*F*(5,12) = 6.392, *P* = 0.004; *F*(5,12) = 10.485, *P* < 0.001, respectively] (*P* < 0.01; *P* < 0.01), and the reductions were partially restored by ICS II at the doses of 8 and 16 mg/kg for 28 days (*P* < 0.05; *P* < 0.01; *P* < 0.05; *P* < 0.01) (Figures 6A–C). BCCAO also remarkably reduced the phosphorylation levels of Akt and CREB [*F*(5,12) = 10.690, *P* < 0.001; *F*(5,12) = 12.946, *P* < 0.001, respectively] (*P* < 0.01; *P* < 0.01), and this reduction was refrained by the treatment of ICS II 8 and 16 mg/kg (*P* < 0.05; *P* < 0.01; *P* < 0.01; *P* < 0.01) (Figures 6A,D,E). These data suggested that ICS II might exert its neuroprotective effect in BCCAO rats by enhancing the BDNF/TrkB/CREB signaling in the hippocampus.

### ICS II Up-Regulated PPARα and PPARγ, but Did Not Alter PPARβ in BCCAO-Treated Rats

**FIGURE 7:**
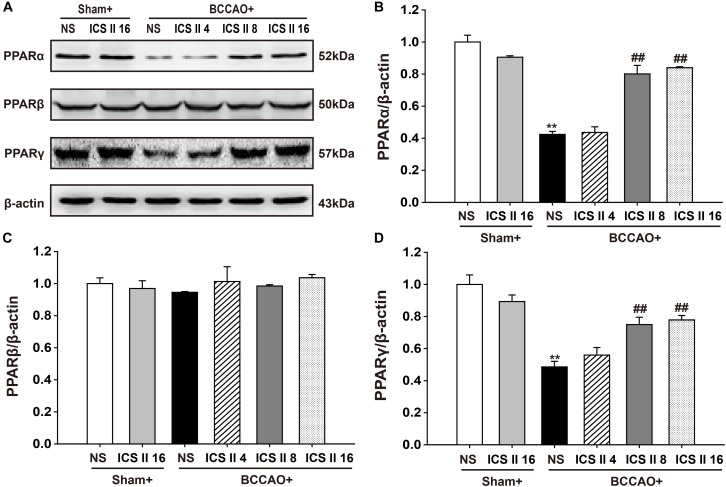
Effect of ICS II on PPARα, PPARγ, and PPARβ in BCCAO-treated rats. The expression levels of PPARα, PPARβ, and PPARγ protein were examined by Western blot. **(A)** The antibody-reactive band of the PPARα, PPARβ, and PPARγ. **(B)** Quantitative analysis of PPARα levels. **(C)** Quantitative analysis of PPARβ levels. **(D)** Quantitative analysis of PPARγ levels. The relative optical density was normalized to β-actin. Data were expressed as mean ± SEM (*n* = 3). ^∗∗^*P* < 0.01 vs. sham, ^##^*P* < 0.01 vs. BCCAO.

To confirm whether the PPARs were associated with the protective effects of ICS II against BCCAO-induced cognitive impairment, the protein expression levels of PPARα, PPARβ, and PPARγ were examined after 28 consecutive days treatment of ICS II. The PPARα and PPARγ levels in BCCAO rats were significantly lower relative to the sham rats [*F*(5,12) = 37.738, *P* < 0.001; *F*(5,12) = 13.323, *P* < 0.001, respectively] (*P* < 0.01; *P* < 0.01). And the PPARα and PPARγ levels in BCCAO + ICS II 8, 16 groups were higher compare to those in BCCAO rats (*P* < 0.01; *P* < 0.01; *P* < 0.01; *P* < 0.01) (Figures 7A,B,D). However, there were no noticeable differences in PPARβ levels among the six groups [*F*(5,12) = 0.498, *P* = 0.772] (Figures 7A,C).

**FIGURE 8:**
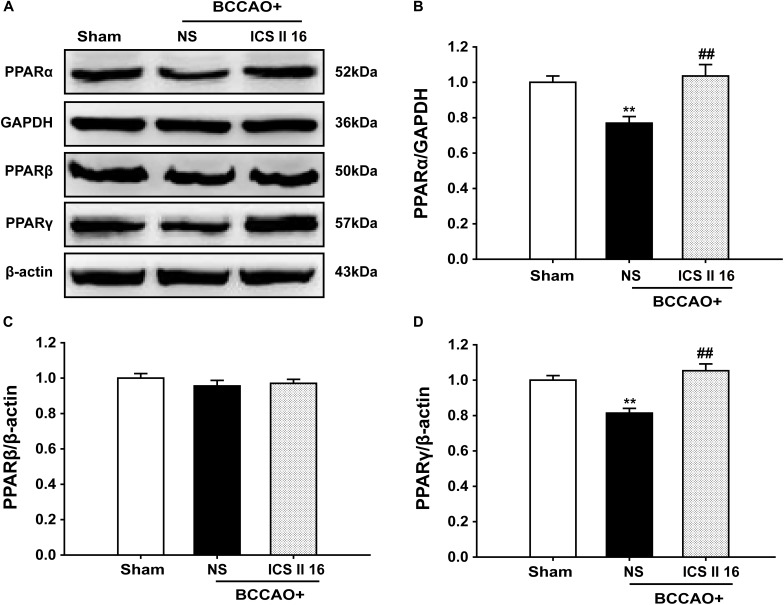
Icariside II up-regulated PPARα and PPARγ, but did not alter PPARβ in BCCAO-treated rats (as the supplemental experiment). The expression levels of PPARα, PPARβ, and PPARγ protein were examined by Western blot. **(A)** The antibody-reactive band of the PPARα, PPARβ, and PPARγ. **(B)** Quantitative analysis of PPARα levels. **(C)** Quantitative analysis of PPARβ levels. **(D)** Quantitative analysis of PPARγ levels. The relative optical density was normalized to GAPDH or β-actin. Data were expressed as mean ± SEM (*n* = 5). ^∗∗^*P* < 0.01 vs. sham, ^##^*P* < 0.01 vs. BCCAO.

It should be noted that the PPARα and PPARγ levels in BCCAO rats that were sacrificed on the 10th day were already decreased compare to sham rats [*F*(2,12) = 9.519, *P* = 0.003; *F*(5,12) = 17.255, *P* < 0.001, respectively] (*P* < 0.01; *P* < 0.01). Interestingly, treatment with ICS II 16 mg/kg for 28 days significantly increased the PPARα and PPARγ expressions as compared to the levels of PPARα and PPARγ in BCCAO rats which were collected after 10 days of surgery (*P* < 0.01; *P* < 0.01) (Figures 8A,B,D). However, there were no significant differences in PPARβ expression among these three groups [*F*(2,12) = 0.727, *P* = 0.504] (Figures 8A,C). These results suggested that ICS II might produce protective effect against BCCAO-induced cognitive impairment by up-regulating the expressions of PPARα and PPARγ, not by retarding the decrease of the expressions of PPARα and PPARγ.

## Discussion

Chronic cerebral hypoperfusion plays a pivotal role in progressive cognitive dysfunction of VD, where it can decrease energy supply to the brain, as well as accelerate neuronal injury and the decline of memory.(Choi et al., 2016; Mohamed et al., 2018). In this study, CCH model induced by BCCAO was applied to investigate the protective effect of ICS II on cognitive impairments, and the results showed that BCCAO could induce spatial learning and memory impairments and neuronal injury in rats, which were consistent with previous reports (Lu et al., 2015; Zhu et al., 2017; Mohamed et al., 2018). Importantly, ICS II effectively and dose-dependently attenuated the spatial learning and memory impairments in BCCAO rats, which might be correlated with the decrease of Aβ_1-40_ and Aβ_1-42_ oligomers, the up-regulation of PPARα and PPARγ, together with the enhancement of BDNF/TrkB/CREB signaling (All the raw data of Western Blot can be found in Supplementary Data Sheet 1).

The presence of amyloid plaques formed by extracellular Aβ deposition is one of the important pathophysiological hallmarks for VD (Sun et al., 2015). The Aβ peptides contain 36–43 amino acid residues in length. In particular, Aβ_1-40_ and Aβ_1-42_ with two additional hydrophobic residues at the carboxy terminus are major constituents of amyloid plaques, where Aβ_1-40_ shows the highest concentration and Aβ_1-42_ possesses the strongest neurotoxicity (Keskitalo et al., 2014; Das and Smid, 2017; Omar, 2017). Furthermore, Aβ_1-42_ is more hydrophobic and less soluble than Aβ_1-40_ (Saxena, 2011). Several studies have reported that the excessive increase of Aβ_1-40_ and Aβ_1-42_ in the hippocampus could reduce synaptic plasticity and disrupt the long-term potentiation (LTP), eventually leading to neuronal loss and memory deficits (Zhu et al., 2015; Yan et al., 2017). Indeed, the secreted Aβ monomers associate into soluble oligomers to form insoluble oligomers, generating protofibrils and fibrils, which can lead to the neurotoxicity via inhibiting LTP, alter the composition and density of synapses, impair synaptic plasticity and spatial memory (Keskitalo et al., 2014; Zhang and Song, 2017). Moreover, there is substantial evidence suggesting that CCH induced by BCCAO abnormally increases Aβ contents, which then facilitates learning and memory deficits (Ai et al., 2013; Zou et al., 2018), and results from our present study are consistent with those findings. Strikingly, prolonged administration of ICS II effectively reduced the levels of Aβ_1-40_ and Aβ_1-42_ soluble oligomers, suggesting that the protective effect of ICS II on BCCAO-induced learning and memory deficits is closely correlated with Aβ reduction.

In the generation of Aβ peptides, APP is a key precursor involved in non-amyloidogenic and amyloidogenic pathways (Pajak et al., 2016). APP in the non-amyloidogenic pathway is catabolized by α-secretase to form the soluble sAPPα fragment and the membrane-anchored 83 amino acid fragment (CTFα or C83), where sAPPα regulates neuronal excitability, enhances synaptic plasticity and improves memory ability, and the C83 is further cleaved by γ-secretase to produce the harmless P3 peptide (Folch et al., 2016). ADAM10 as the most active α-secretase competes with BACE1 in the APP processing to inhibit Aβ generation and reduce the formation of plaque, exerts neurotrophic and neuroprotective functions (Vassar, 2013; Andrew et al., 2016). Our data showed that BCCAO prevented the protein expression of ADAM10, thereby leading to the increase of Aβ levels *in vivo*. Importantly, the low ADAM10 protein in BCCAO rats was increased by treatment with ICS II for 28 days. On the other hand, the amyloidogenic pathway starts with the process that APP in the N-terminal of Aβ domain is cleaved by β-secretase (BACE1, a rate limiting enzyme of Aβ generation) to secrete the soluble sAPPβ and the membrane-anchored 99 amino acid fragments (CTFβ or C99), and then the cleavage of the C99 fragment by γ-secretase leads to the production of Aβ peptides (Laitera et al., 2015). Importantly, the amyloidogenic pathway occurs in a neuropathological situation, and the producted Aβ peptides can form extracellular Aβ fibrils which may cause the reduction of neuronal plasticity and synaptic loss, even trigger neurotoxicity and neurodegeneration (Folch et al., 2016; Fulop et al., 2018). BACE1, a membrane-bound aspartic protease, is involved in the early stage of APP processing (Ghosh and Osswald, 2014). Moreover, previous studies have reported that inhibiting BACE1 activity causes the decrease of APP cleavage to prevent sAPPβ formation, which then down-regulates Aβ levels (Thakker et al., 2015). Interestingly, similar to previous observations, ICS II treatment for 28 days clearly decreased BCCAO-induced expression of APP and BACE1, leading to the reduction of Aβ levels in VD, indicating the prevention of the amyloidogenic pathway in APP processing is benefit for the inhibition of Aβ generation in the neuropathological mode of CCH. These data suggested that the protective effect of ICS II against BCCAO-induced learning and memory deficits was associated with the inhibition of Aβ generation mediated by both the up-regulation of ADAM10 and the suppression of APP and BACE1.

The insufficient Aβ degradation could lead to the accumulation of Aβ, and disruption of cerebral homeostasis of Aβ (Del Campo et al., 2015; Mohamed et al., 2016). The insulin degrading enzyme (IDE) is considered one of the most important Aβ-degrading enzymes in the hippocampus, which is studied extensively in the degradation of extracellular Aβ. Recently, the increase of endogenous Aβ in the brain was observed in the IDE knockout mice (Pivovarova et al., 2016). Furthermore, IDE overexpression in neurons decreased cerebral Aβ levels and prevented the formation of Aβ plaque (Guo et al., 2015). The present study found that the expression of IDE in BCCAO rats was significantly decreased, consistent with a previous report (Li W. X. et al., 2015). Importantly, ICS II restored the reduction of IDE in the hippocampus of BCCAO rats, which possibly promoted Aβ degradation and then exerted its anti-cognitive deficits effects. In addition, neprilysin (NEP) is also an important Aβ-degrading enzyme, which cannot only degrade extracellular Aβ oligomers, but also destroy the functional domains of APP in brain, directly ameliorating the toxicity of Aβ to the central nervous system (Cai et al., 2017). Studies revealed the decline of NEP activity could excessively increase Aβ levels, and intracranial injection of NEP caused an acute reduction in Aβ levels (Chang et al., 2015; Becker et al., 2018). Importantly, previous evidence has shown that ICS II could increase NEP expression to exert the neuroprotective effect in STZ-induced AD rats (Yin et al., 2016). Thus, we concluded that ICS II could concurrently enhance the activity of IDE and NEP, and then ameliorate cognitive deficits, eventually exerting the neuroprotective effect in BCCAO-induced VD rats.

There is substantial evidence that BDNF is a key regulator in synaptic plasticity, neuronal survival and learning and memory, which binds to its high-affinity receptor TrkB and then causes Akt phosphorylation to exert neuroprotective effect on ischemic brain injury (Ai et al., 2013; Xu et al., 2015). Moreover, the activation of BDNF/TrkB signaling can enhance the LTP of hippocampal synapses and improve spatial memory (Tanila, 2017). The possible mechanism is associated with the up-regulation of CREB phosphorylation (Wu et al., 2015). CREB is an important nuclear transcription factor that regulates the transcription of BDNF and affects the processes of learning and memory by its phosphorylation resulting from the activation of Akt signaling (Rosa and Fahnestock, 2015; Zhou et al., 2016). However, oligomeric Aβ can induce the reduction of CREB phosphorylation, and then down-regulate BDNF levels, eventually leading to spatial memory dysfunction (Tanila, 2017). In addition, recent studies showed soluble Aβ reduced BDNF expression, and restoring the reduction of BDNF content could limit the neurotoxicity of Aβ oligomers (Schiavone et al., 2017; Morgese et al., 2018). CCH could decrease BDNF expression and increase Aβ generation, while the increase of Aβ further promoted the down-regulation of BDNF (Li et al., 2016). Hence, it was speculated that an increase of Aβ induced by BCCAO might block the BDNF/TrkB/CREB signaling. The present study confirmed the speculation that the expression of BDNF and TrkB together with phosphorylation of Akt and CREB were reduced in the hippocampus of BCCAO rats. ICS II reversed the reduction of BDNF and TrkB expression, and restored the low levels of Akt and CREB phosphorylation. These findings indicated that ICS II exerted a neuroprotective effect on BCCAO-induced cognitive deficits in rats, which was due to the enhancement of BDNF/TrkB/CREB signaling. In fact, BDNF and nerve growth factor (NGF) are all belong to neurotrophic factor protein. As we known, BDNF can bind to TrkB to exert neuroprotective effect on ischemic brain injury. While NGF can regulate the growth of peripheral and central neurons, maintain neuronal survival and synapse structure stability, which binds to its high-affinity receptor TrkA to ameliorate progressive cognitive deficits, exerting the neuroprotective effect in AD (Canu et al., 2017). Furthermore, studies found that BDNF and NGF could inhibit neuroinflammation by modulating nuclear factor-κB (NF-κB) (Cai et al., 2017). Interestingly, previous evidences showed that ICS II could relieve neuroinflammation by suppressing NF-κB activation to exert the neuroprotective effect on middle cerebral artery occlusion (MCAO)-induced cognitive deficits in rats (Deng et al., 2016). Hence, we speculated that ICS II might concurrently enhance BDNF/TrkB signaling and NGF/TrkA signaling to modulate neuroinflammation, eventually exerting the neuroprotective effect in BCCAO-induced VD rats, which will be confirmed in our further study. For various therapeutic targets in several neurodegenerative disorders such as AD, Parkinson disease and Huntington disease, the nuclear receptor peroxisome proliferator-activated receptors (PPARs) have recently gained much attention (Agarwal et al., 2017). PPARs family members contain PPARα, PPARβ/δ, and PPARγ that belong to ligand-activated transcription factors controlling the gene expression (Agarwal et al., 2017). Numerous studies have shown that the protective effects of PPARα and PPARγ on AD model were associated with the reduction of Aβ levels, where PPARα stimulated APP degradation by up-regulating the transcription of α-secretase (ADAM10) to produce sAPPα and decrease Aβ production, while PPARγ modulated the clearance of Aβ through the inhibition of β-secretase (BACE1) transcription (Zhang et al., 2014; Yan et al., 2017). Interestingly, our findings found that the PPARα and PPARγ levels in BCCAO rats were significantly lower compared to the sham rats, while the PPARα and PPARγ levels in BCCAO + ICS II 8, 16 groups were higher than those in BCCAO rats, but there were no noticeable differences in PPARβ levels among the six groups. The results suggested that the protective effect of ICS II on BCCAO rats might be mediated by PPARα and PPARγ, but it was not clear whether ICS II retarded the decrease of the expressions of PPARα and PPARγ or ICS II up-regulated the expressions of PPARα and PPARγ. To clarify the problem and get a better perspective, another experiment was performed, and the results found that the protein expression of PPARα and PPARγ in BCCAO rats sacrificed on the 10th day were lower than those of the sham rats, while treatment with ICS II for 28 days, the protein expression of PPARα and PPARγ exhibited higher level. The data clearly demonstrated that ICS II might produce protective effect against BCCAO-induced cognitive impairment by up-regulating the expressions of PPARα and PPARγ, not by retarding the decrease of the expressions of PPARα and PPARγ.

In summary, the present study clearly demonstrated that ICS II can attenuate BCCAO-induced cognitive deficits and hippocampal neuronal death by suppressing Aβ generation and promoting Aβ degradation. The effect of ICS II on Aβ reduction is mediated by up-regulation of PPARα and PPARγ, and the activation of BDNF/TrkB/CREB signaling. These findings confirm for the first time that ICS II is a promising candidate to treat dementia induced by CCH such as VD.

## Author Contributions

CY, YD, YL, JG, LY, and QG performed the study. CY, YD, and QG wrote the manuscript.

## Conflict of Interest Statement

The authors declare that the research was conducted in the absence of any commercial or financial relationships that could be construed as a potential conflict of interest.
