# Supplementary figures and images for: Icariside II Ameliorates Cognitive Impairments Induced by Chronic Cerebral Hypoperfusion by Inhibiting the Amyloidogenic Pathway: Involvement of BDNF/TrkB/CREB Signaling and Up-Regulation of PPARα and PPARγ in Rats
Source: Front Pharmacol. 2018 Oct 23;9:1211. doi: 10.3389/fphar.2018.01211 (PMC6206175; doi:10.3389/fphar.2018.01211)

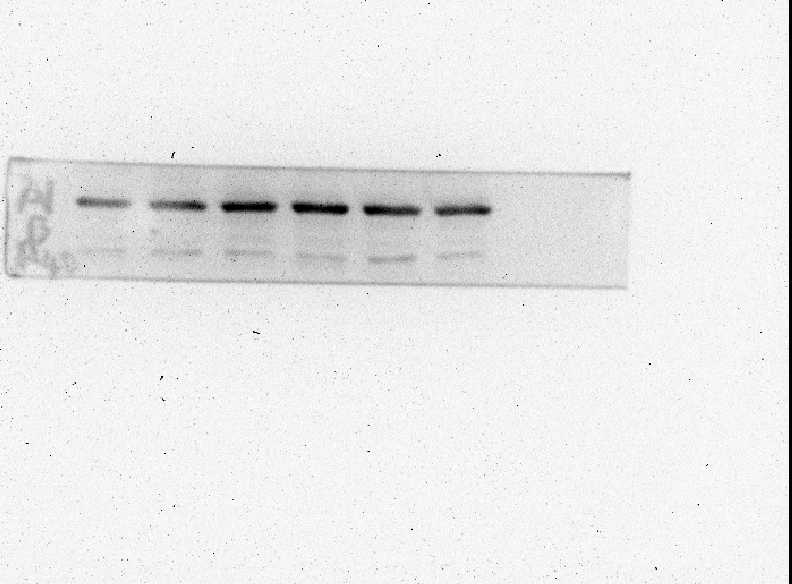

Supplement: Supplementary file 1 [file Data_Sheet_1.ZIP › the file of original Western Blot/figure4/Aa┬1-40/Aa┬1-40-1.jpg]

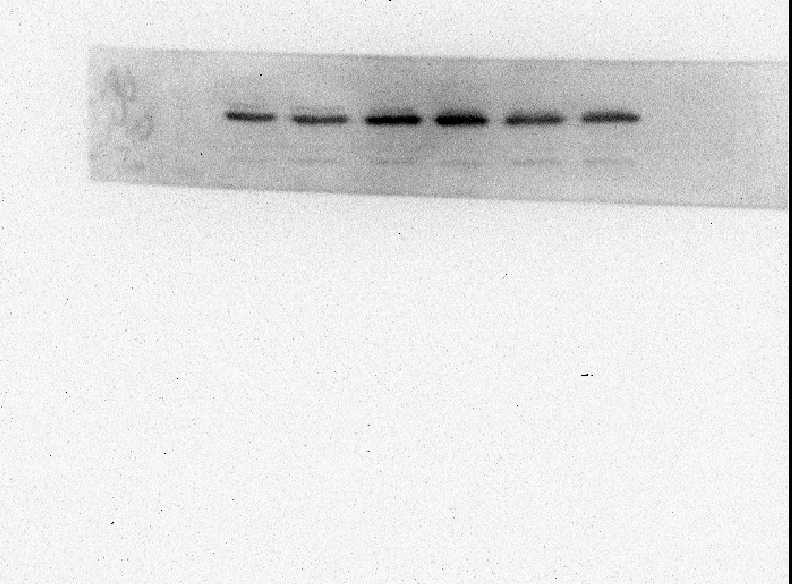

Supplement: Supplementary file 1 [file Data_Sheet_1.ZIP › the file of original Western Blot/figure4/Aa┬1-40/Aa┬1-40-2.jpg]

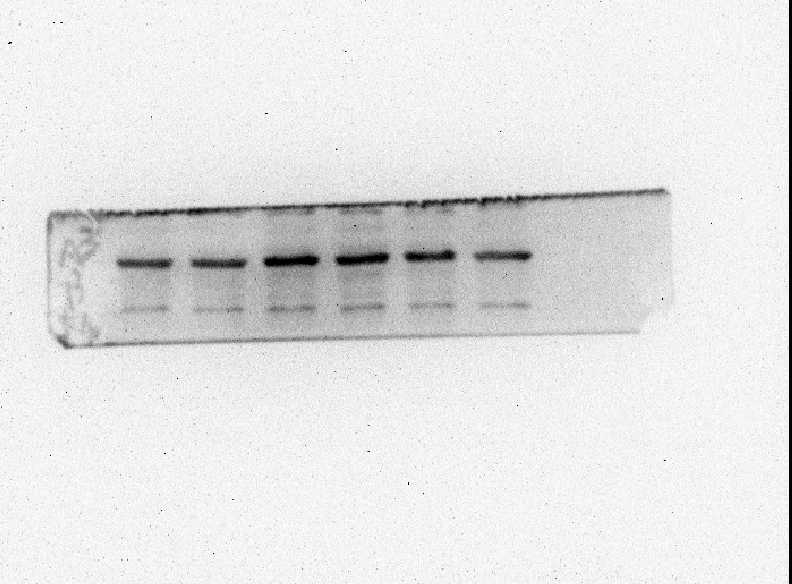

Supplement: Supplementary file 1 [file Data_Sheet_1.ZIP › the file of original Western Blot/figure4/Aa┬1-40/Aa┬1-40-3.jpg]

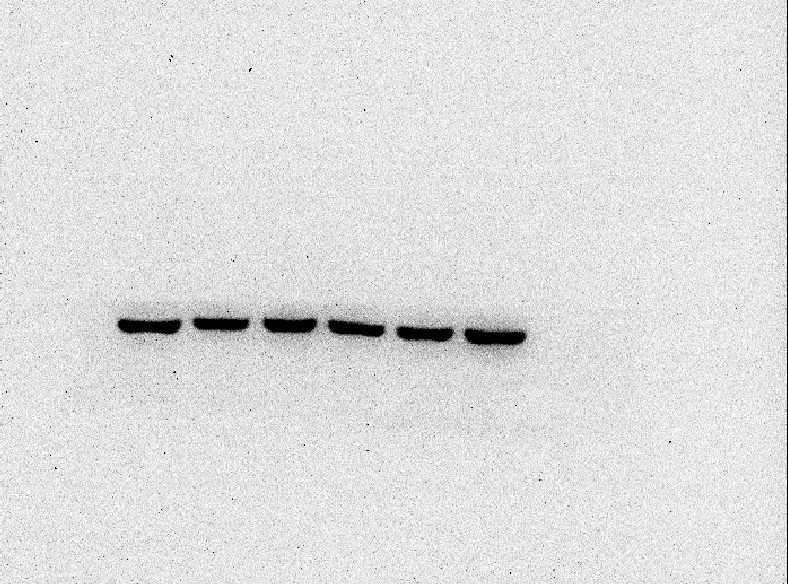

Supplement: Supplementary file 1 [file Data_Sheet_1.ZIP › the file of original Western Blot/figure4/Aa┬1-40/a┬-actin-1.jpg]

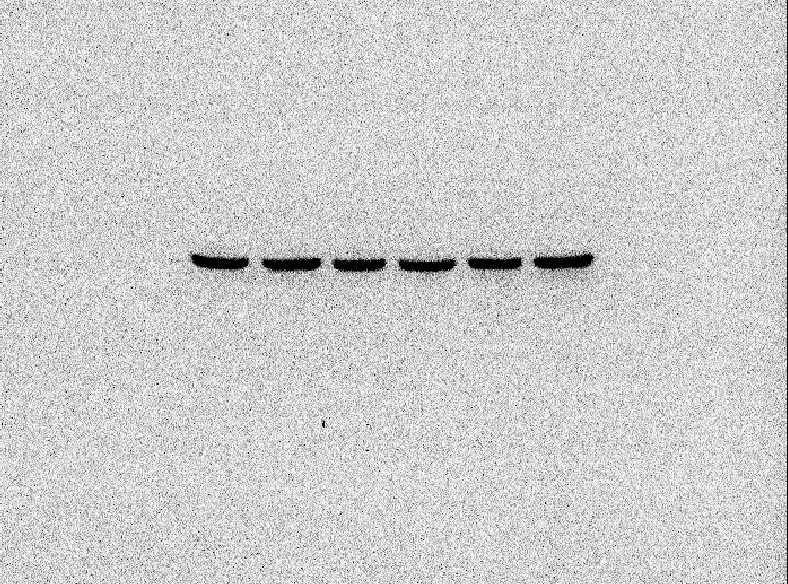

Supplement: Supplementary file 1 [file Data_Sheet_1.ZIP › the file of original Western Blot/figure4/Aa┬1-40/a┬-actin-2.jpg]

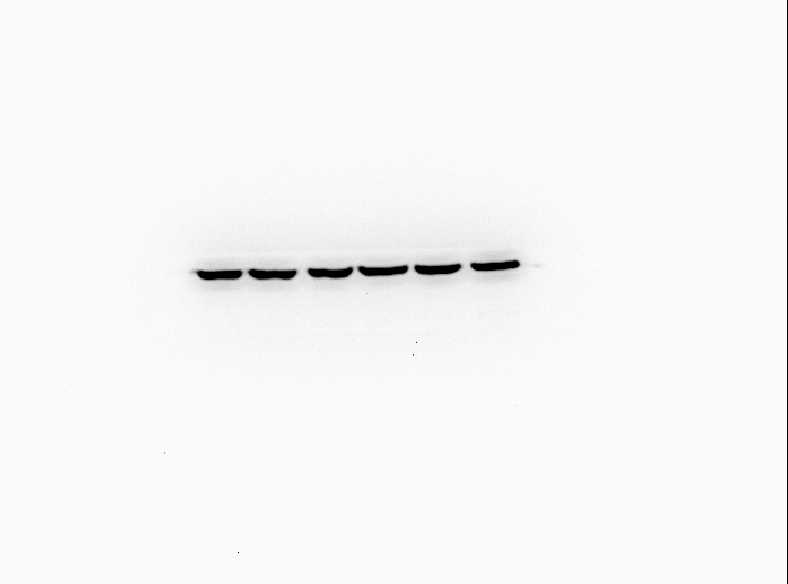

Supplement: Supplementary file 1 [file Data_Sheet_1.ZIP › the file of original Western Blot/figure4/Aa┬1-40/a┬-actin-3.jpg]

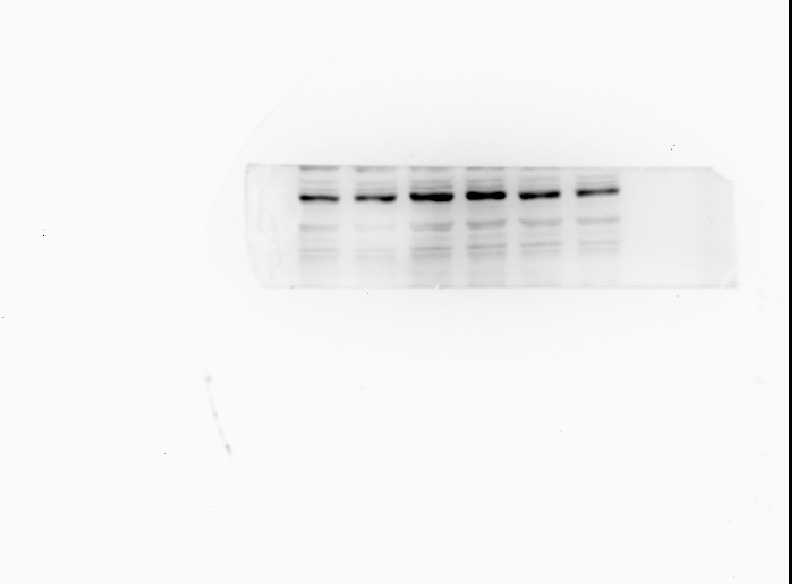

Supplement: Supplementary file 1 [file Data_Sheet_1.ZIP › the file of original Western Blot/figure4/Aa┬1-42/Aa┬1-42-1.jpg]

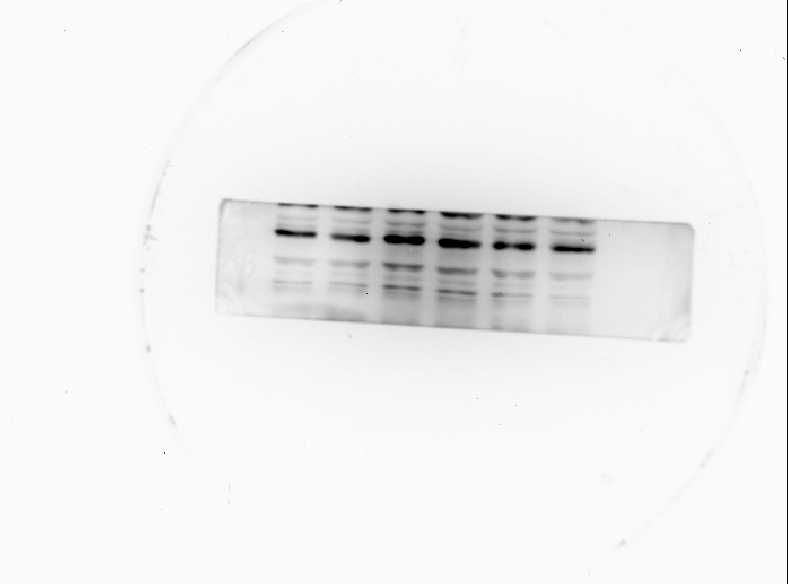

Supplement: Supplementary file 1 [file Data_Sheet_1.ZIP › the file of original Western Blot/figure4/Aa┬1-42/Aa┬1-42-2.jpg]

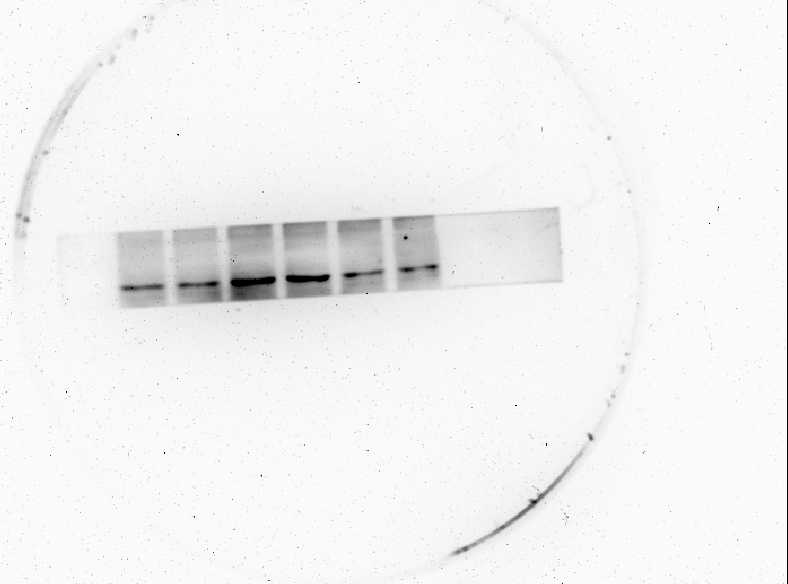

Supplement: Supplementary file 1 [file Data_Sheet_1.ZIP › the file of original Western Blot/figure4/Aa┬1-42/Aa┬1-42-3.jpg]

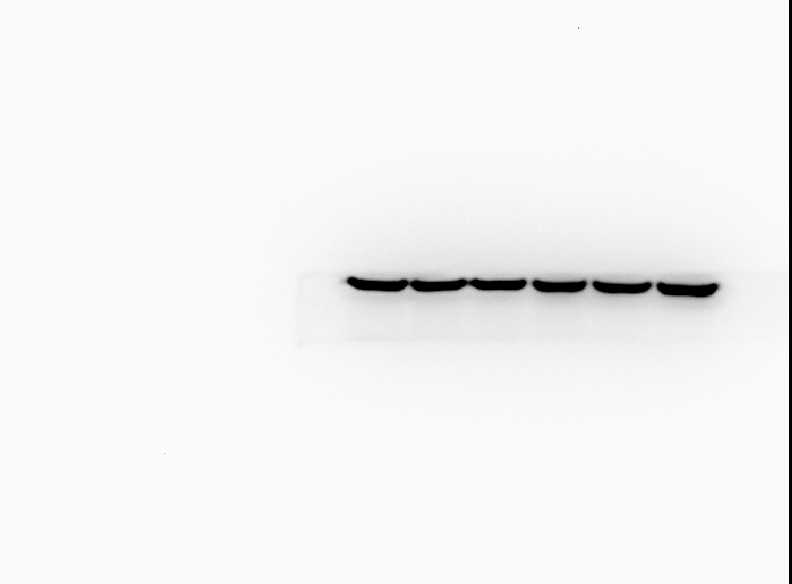

Supplement: Supplementary file 1 [file Data_Sheet_1.ZIP › the file of original Western Blot/figure4/Aa┬1-42/a┬-actin-1.jpg]

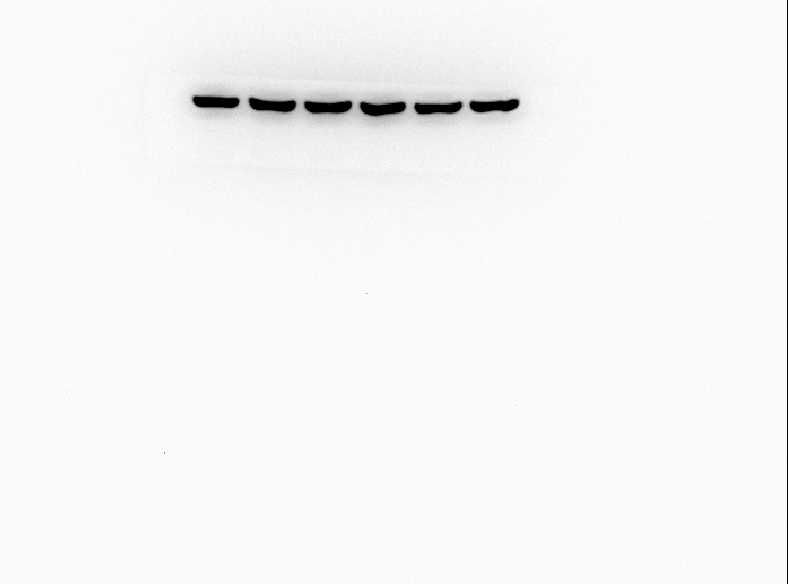

Supplement: Supplementary file 1 [file Data_Sheet_1.ZIP › the file of original Western Blot/figure4/Aa┬1-42/a┬-actin-2.jpg]

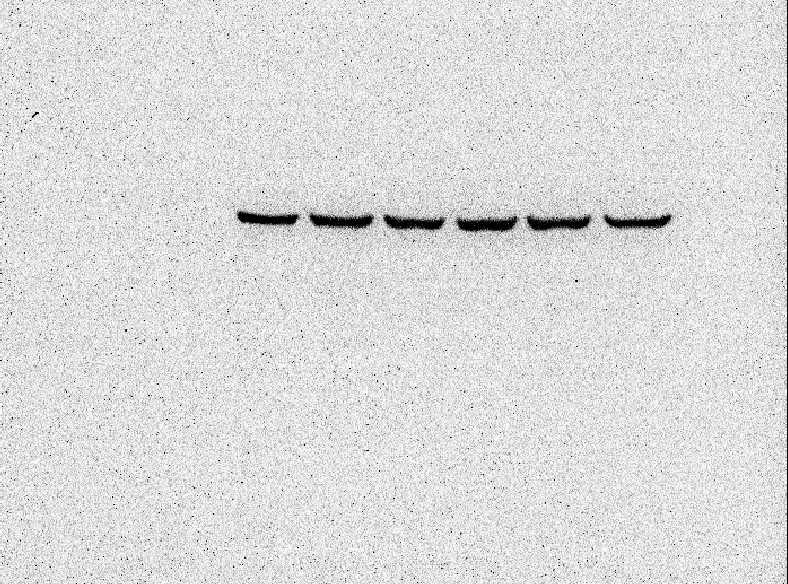

Supplement: Supplementary file 1 [file Data_Sheet_1.ZIP › the file of original Western Blot/figure4/Aa┬1-42/a┬-actin-3.jpg]

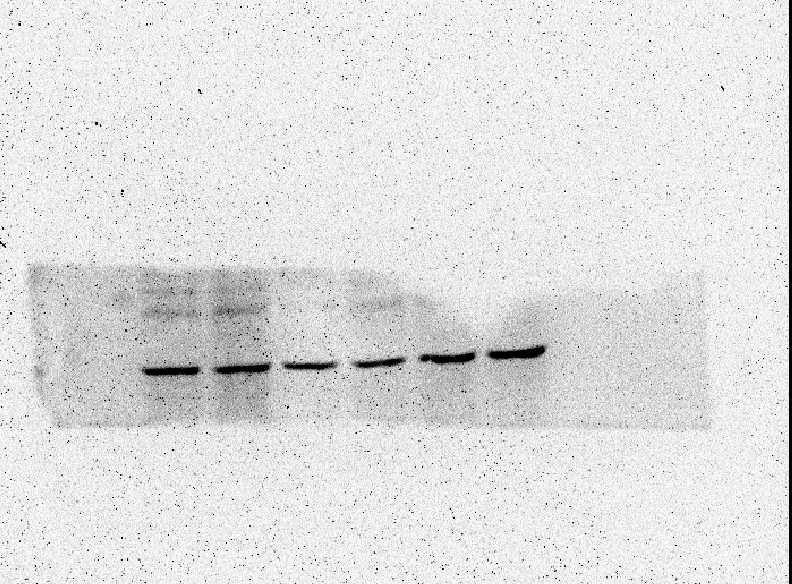

Supplement: Supplementary file 1 [file Data_Sheet_1.ZIP › the file of original Western Blot/figure5/ADAM10/ADAM10-1.jpg]

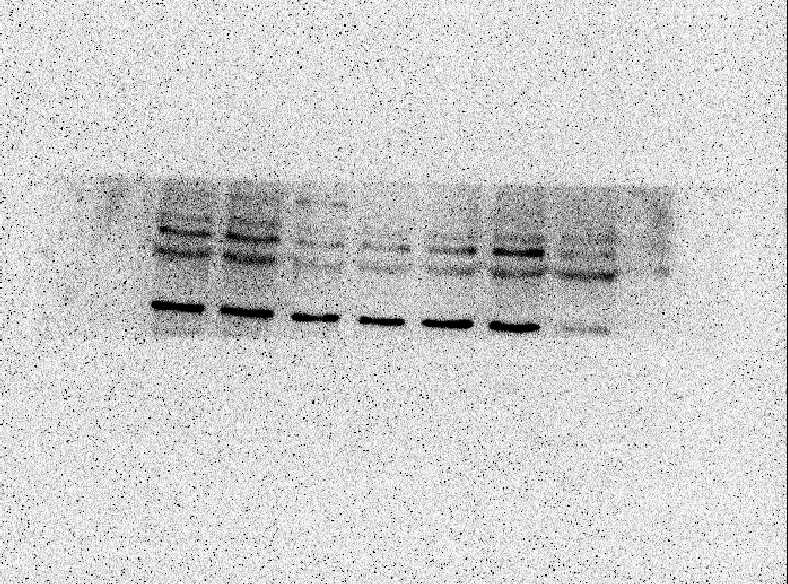

Supplement: Supplementary file 1 [file Data_Sheet_1.ZIP › the file of original Western Blot/figure5/ADAM10/ADAM10-2.jpg]

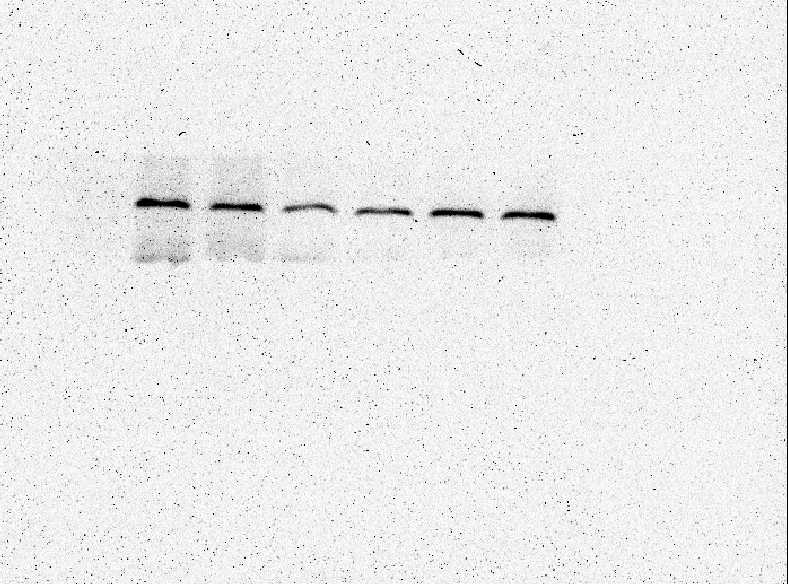

Supplement: Supplementary file 1 [file Data_Sheet_1.ZIP › the file of original Western Blot/figure5/ADAM10/ADAM10-3.jpg]

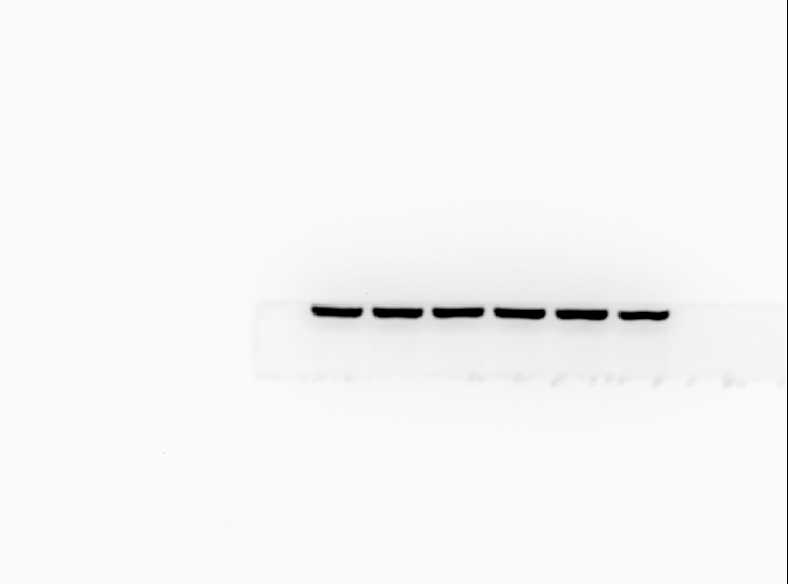

Supplement: Supplementary file 1 [file Data_Sheet_1.ZIP › the file of original Western Blot/figure5/ADAM10/a┬-actin-1.jpg]

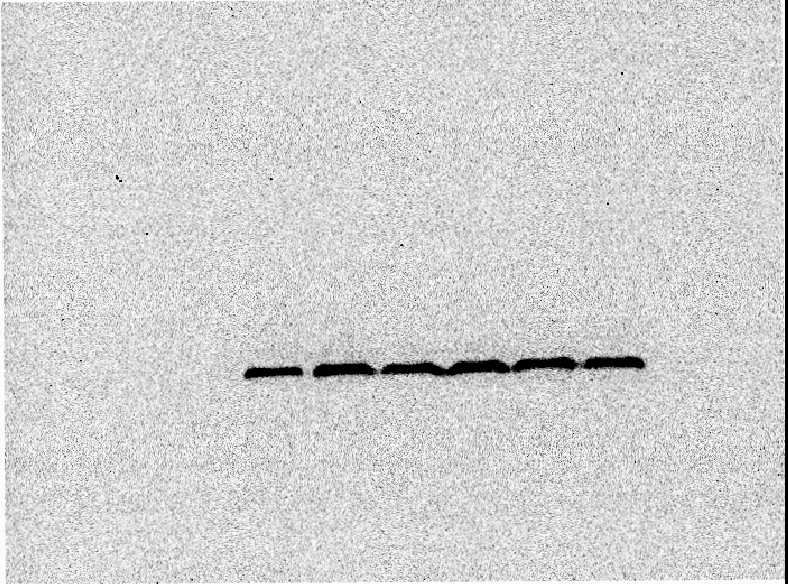

Supplement: Supplementary file 1 [file Data_Sheet_1.ZIP › the file of original Western Blot/figure5/ADAM10/a┬-actin-2.jpg]

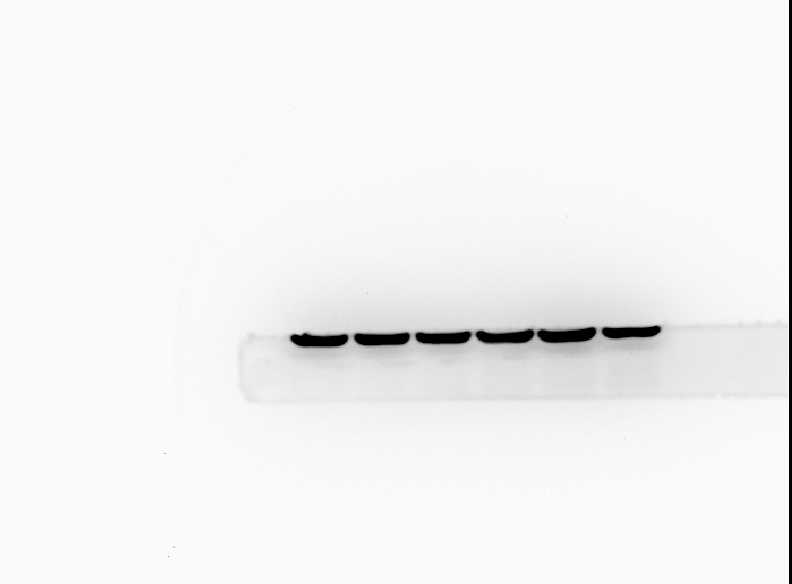

Supplement: Supplementary file 1 [file Data_Sheet_1.ZIP › the file of original Western Blot/figure5/ADAM10/a┬-actin-3.jpg]

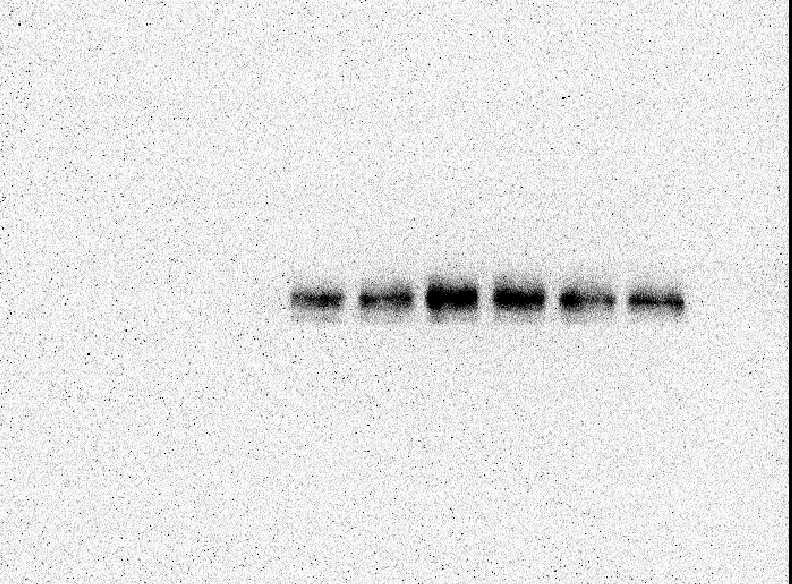

Supplement: Supplementary file 1 [file Data_Sheet_1.ZIP › the file of original Western Blot/figure5/APP/APP-1.jpg]

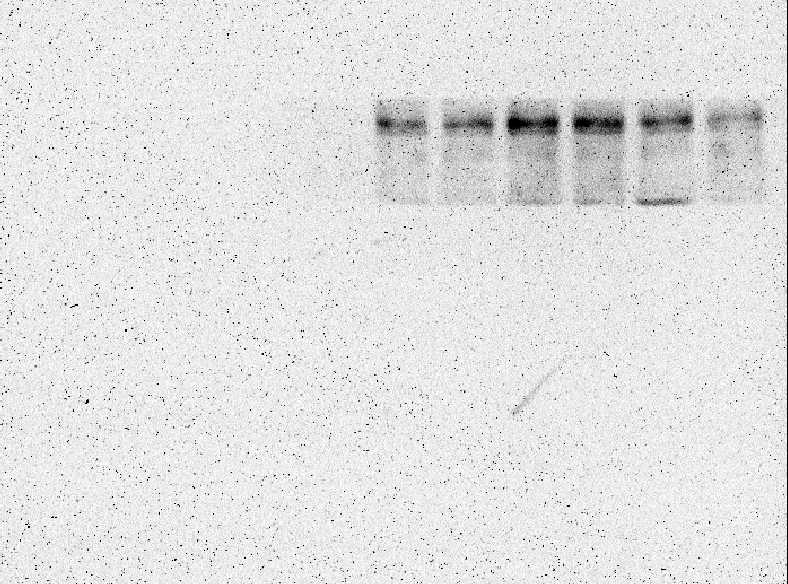

Supplement: Supplementary file 1 [file Data_Sheet_1.ZIP › the file of original Western Blot/figure5/APP/APP-2.jpg]

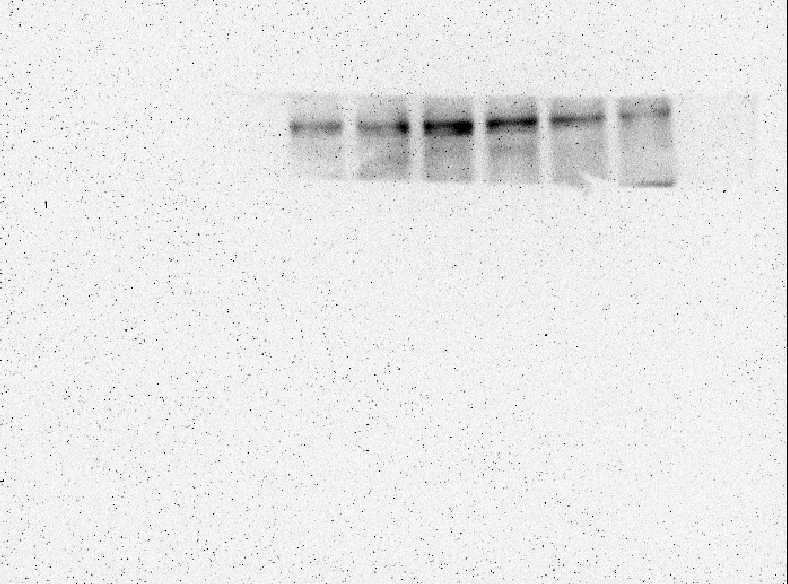

Supplement: Supplementary file 1 [file Data_Sheet_1.ZIP › the file of original Western Blot/figure5/APP/APP-3.jpg]

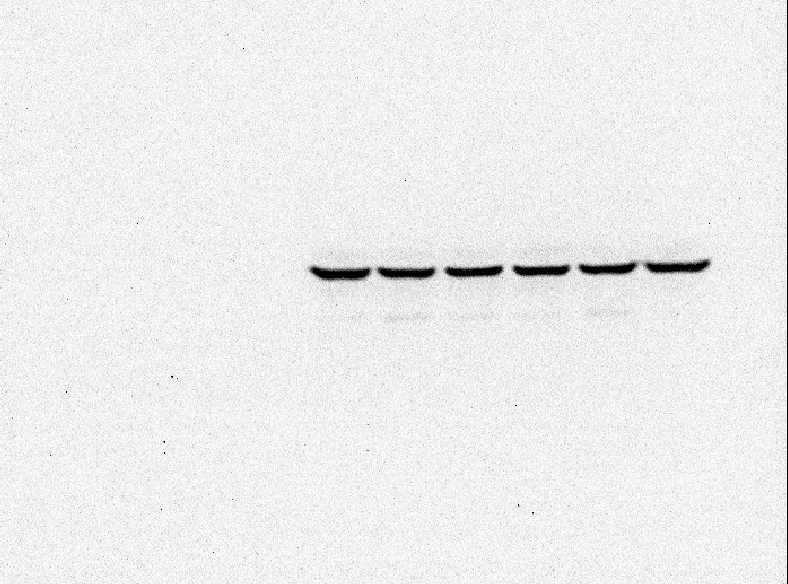

Supplement: Supplementary file 1 [file Data_Sheet_1.ZIP › the file of original Western Blot/figure5/APP/a┬-actin-1.jpg]

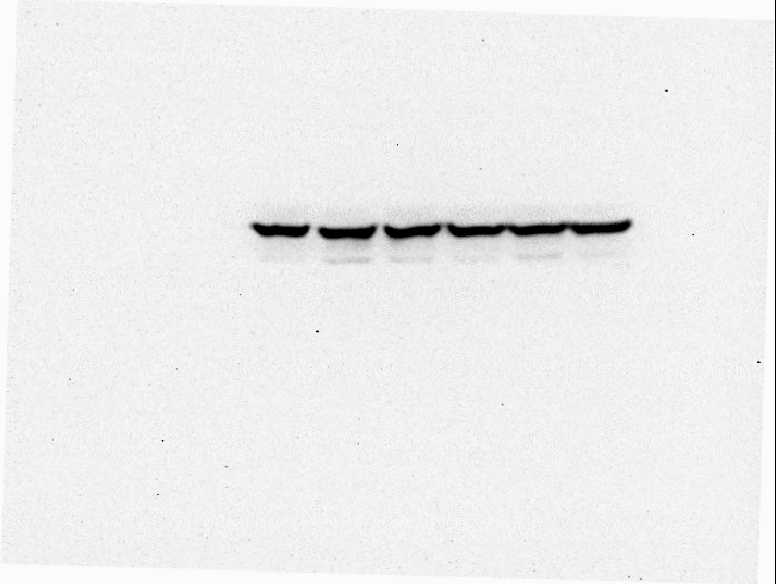

Supplement: Supplementary file 1 [file Data_Sheet_1.ZIP › the file of original Western Blot/figure5/APP/a┬-actin-2.jpg]

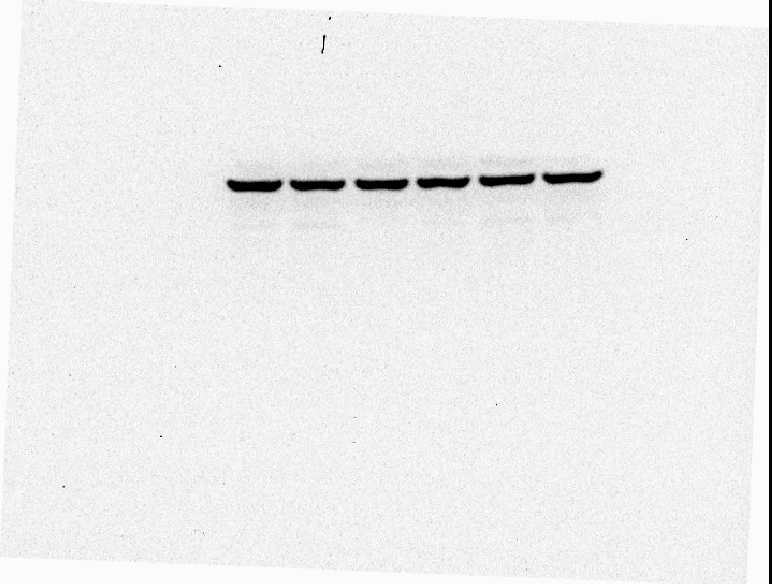

Supplement: Supplementary file 1 [file Data_Sheet_1.ZIP › the file of original Western Blot/figure5/APP/a┬-actin-3.jpg]

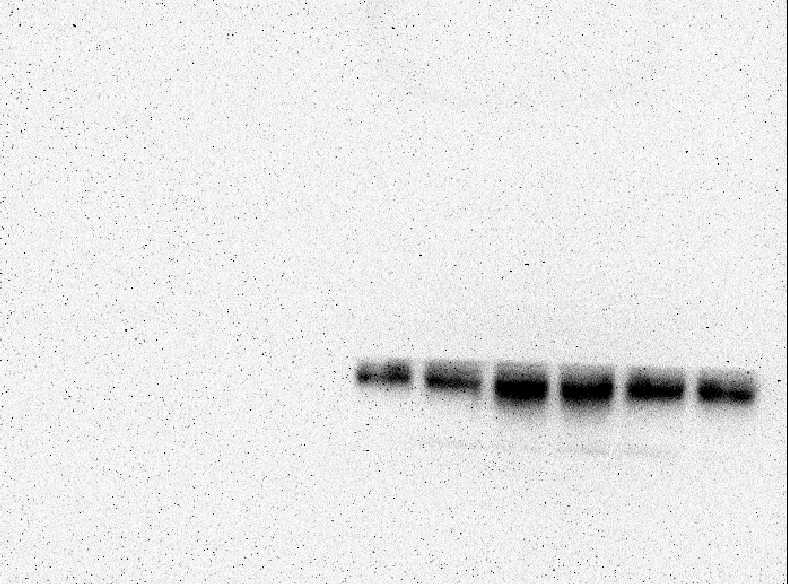

Supplement: Supplementary file 1 [file Data_Sheet_1.ZIP › the file of original Western Blot/figure5/BACE1/BACE1-1.jpg]

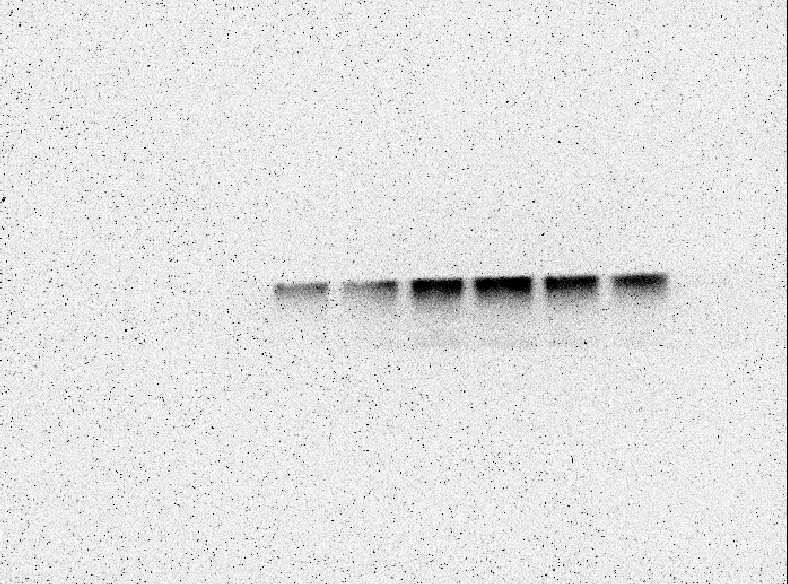

Supplement: Supplementary file 1 [file Data_Sheet_1.ZIP › the file of original Western Blot/figure5/BACE1/BACE1-2.jpg]

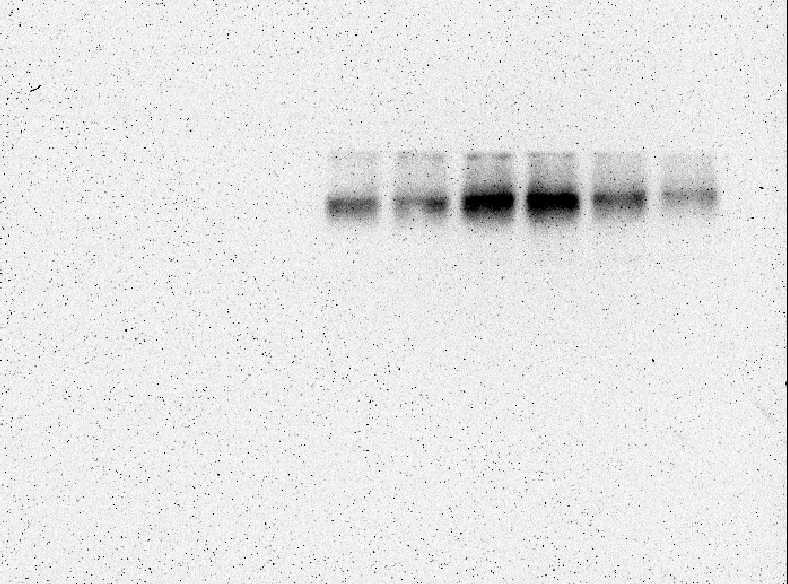

Supplement: Supplementary file 1 [file Data_Sheet_1.ZIP › the file of original Western Blot/figure5/BACE1/BACE1-3.jpg]

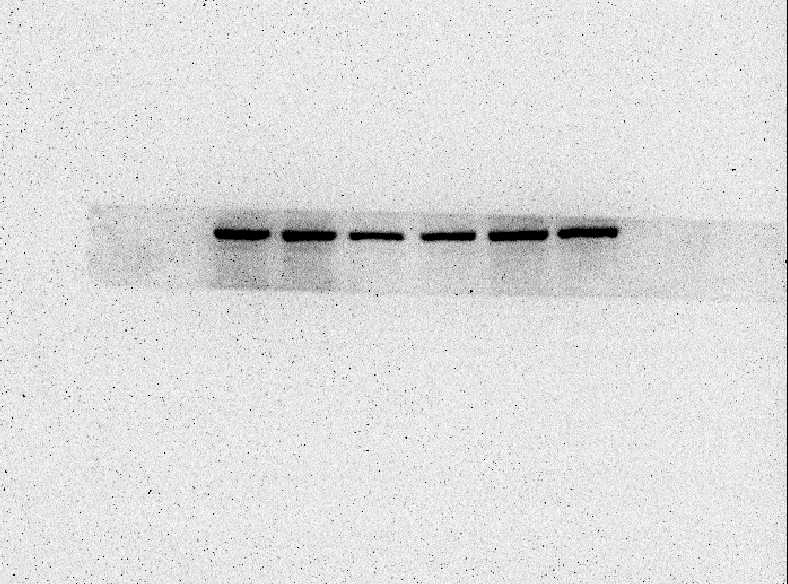

Supplement: Supplementary file 1 [file Data_Sheet_1.ZIP › the file of original Western Blot/figure5/IDE/IDE-1.jpg]

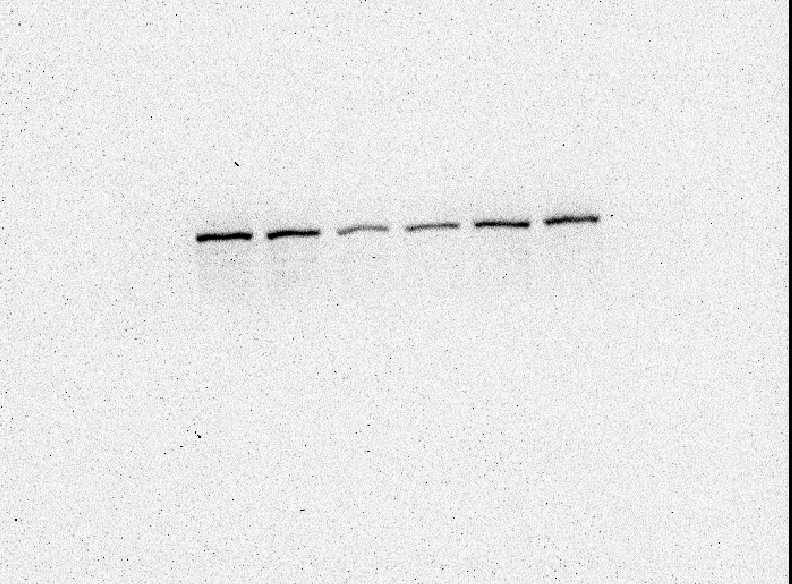

Supplement: Supplementary file 1 [file Data_Sheet_1.ZIP › the file of original Western Blot/figure5/IDE/IDE-2.jpg]

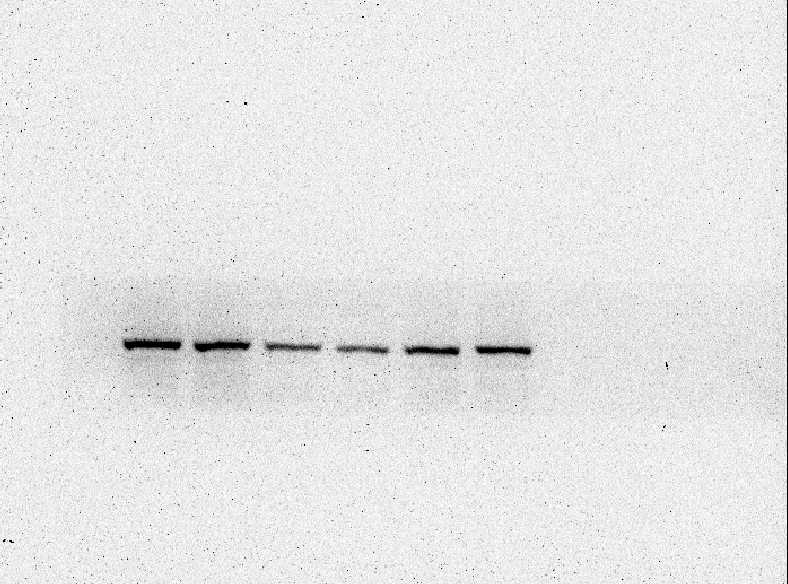

Supplement: Supplementary file 1 [file Data_Sheet_1.ZIP › the file of original Western Blot/figure5/IDE/IDE-3.jpg]

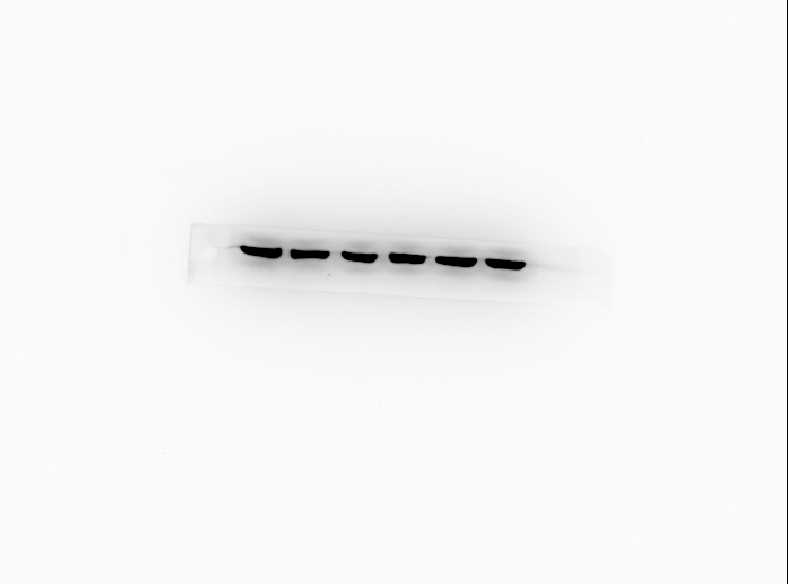

Supplement: Supplementary file 1 [file Data_Sheet_1.ZIP › the file of original Western Blot/figure5/IDE/a┬-actin-1.jpg]

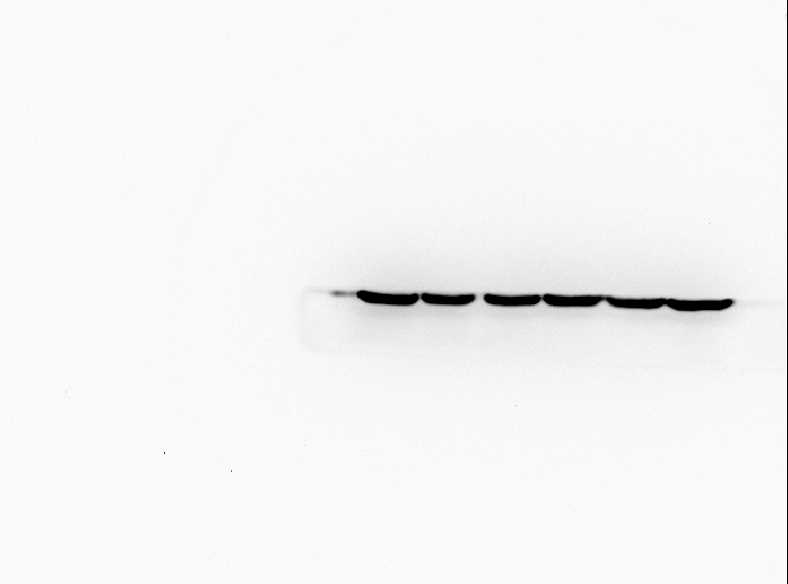

Supplement: Supplementary file 1 [file Data_Sheet_1.ZIP › the file of original Western Blot/figure5/IDE/a┬-actin-2.jpg]

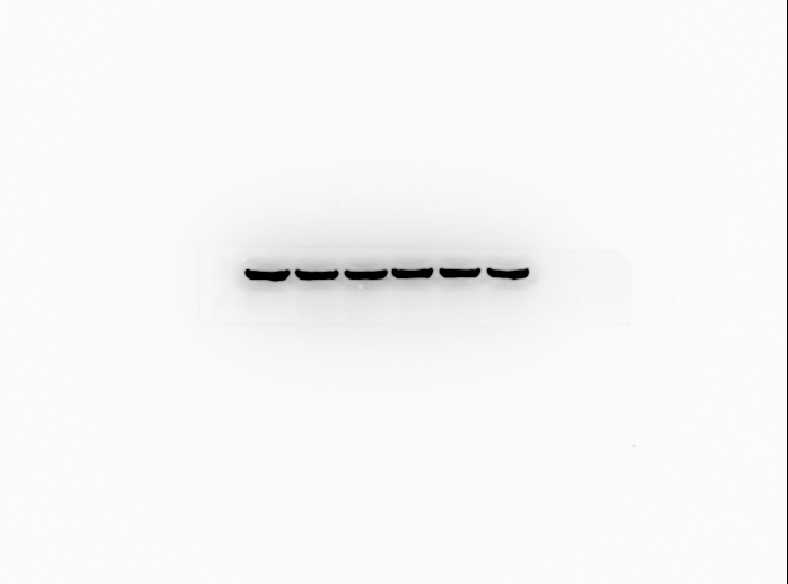

Supplement: Supplementary file 1 [file Data_Sheet_1.ZIP › the file of original Western Blot/figure5/IDE/a┬-actin-3.jpg]

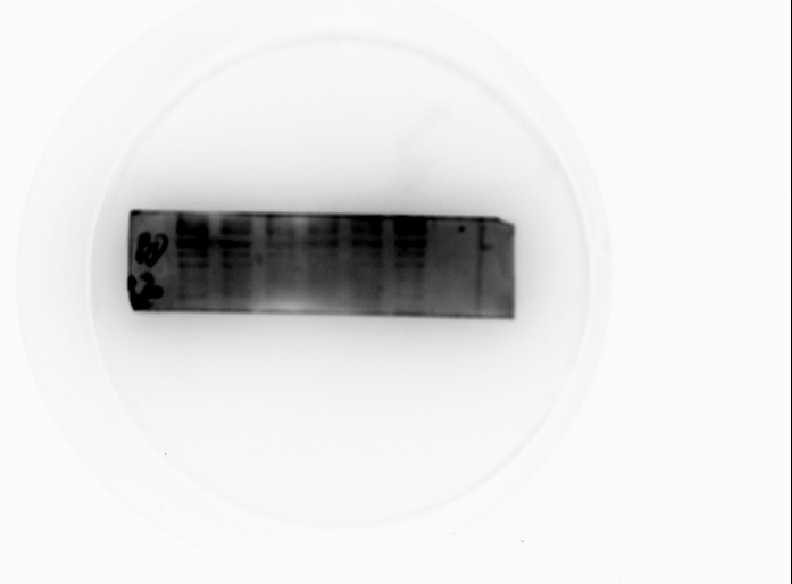

Supplement: Supplementary file 1 [file Data_Sheet_1.ZIP › the file of original Western Blot/figure6/BDNF/BDNF-1.jpg]

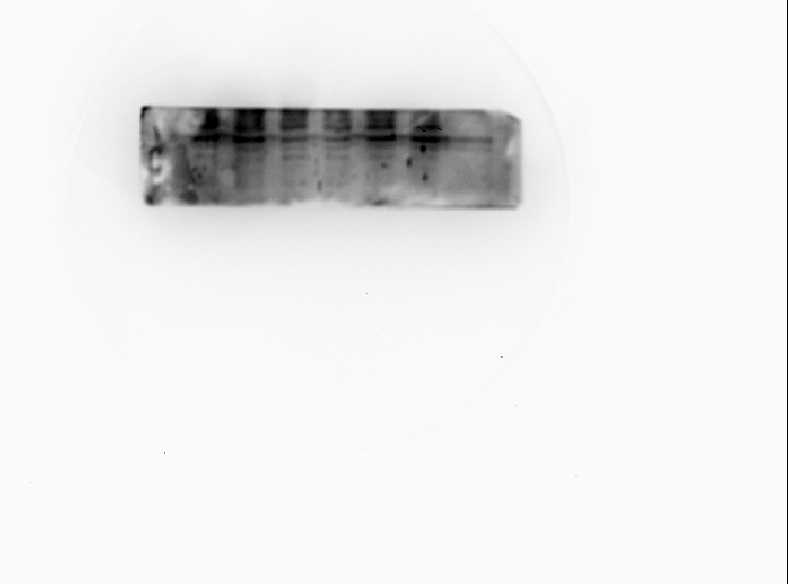

Supplement: Supplementary file 1 [file Data_Sheet_1.ZIP › the file of original Western Blot/figure6/BDNF/BDNF-2.jpg]

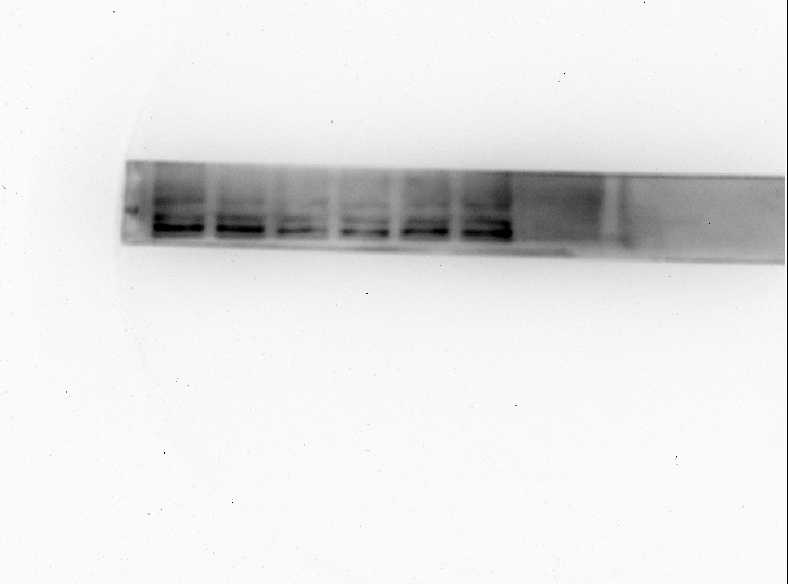

Supplement: Supplementary file 1 [file Data_Sheet_1.ZIP › the file of original Western Blot/figure6/BDNF/BDNF-3.jpg]

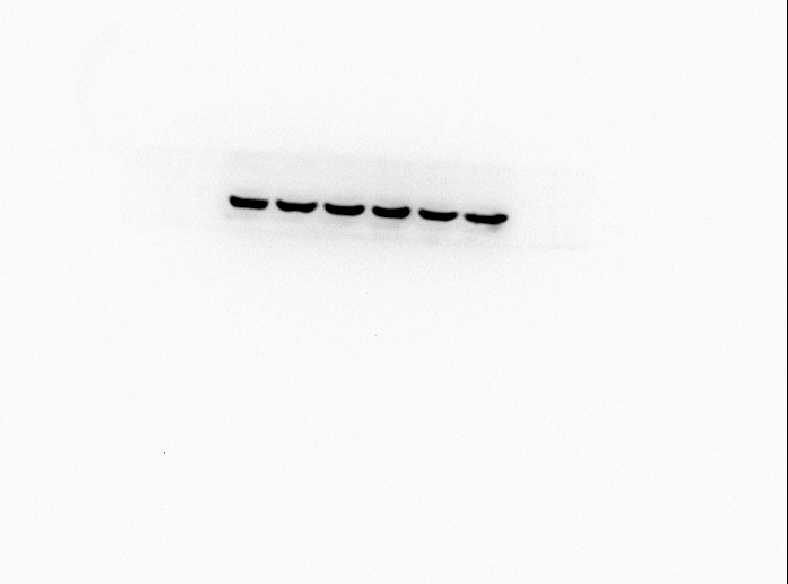

Supplement: Supplementary file 1 [file Data_Sheet_1.ZIP › the file of original Western Blot/figure6/BDNF/a┬-actin-1.jpg]

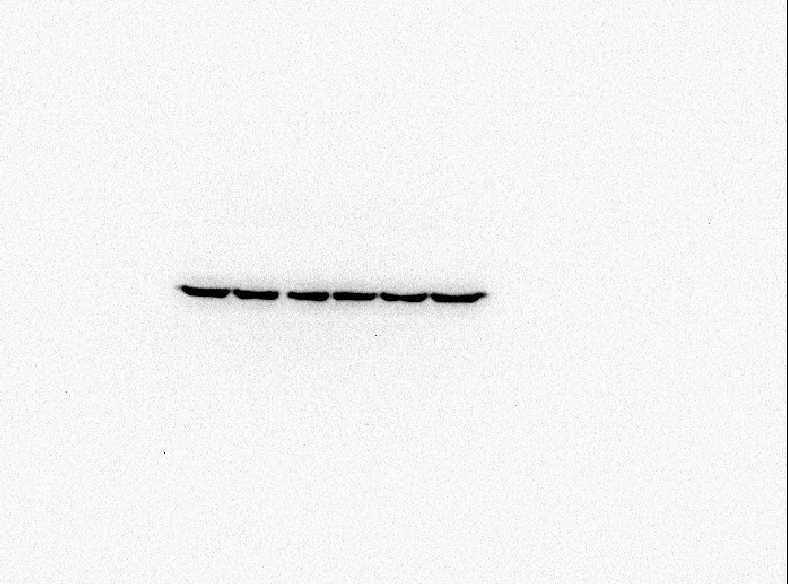

Supplement: Supplementary file 1 [file Data_Sheet_1.ZIP › the file of original Western Blot/figure6/BDNF/a┬-actin-2.jpg]

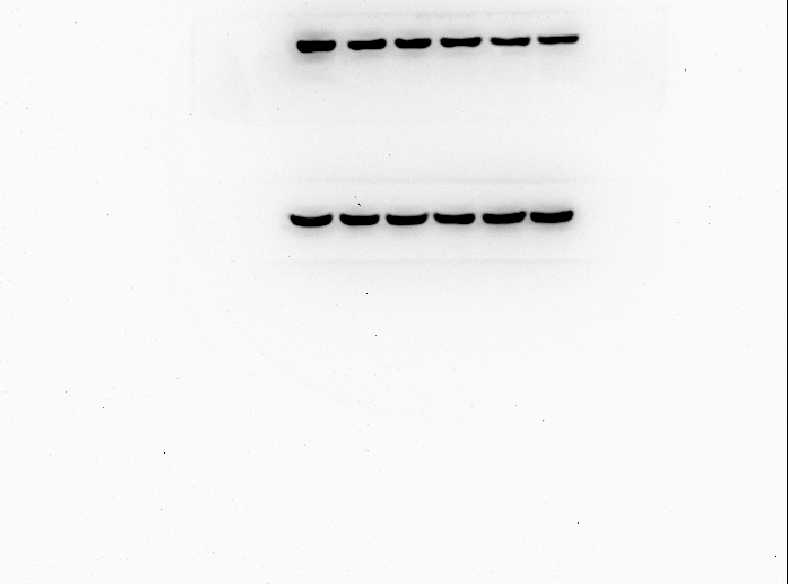

Supplement: Supplementary file 1 [file Data_Sheet_1.ZIP › the file of original Western Blot/figure6/BDNF/a┬-actin-3.jpg]

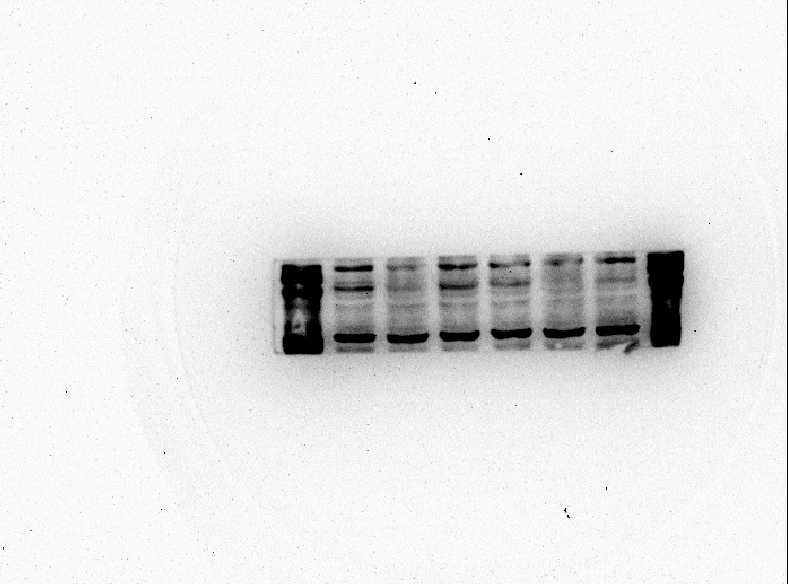

Supplement: Supplementary file 1 [file Data_Sheet_1.ZIP › the file of original Western Blot/figure6/p-Akt/Akt-1.jpg]

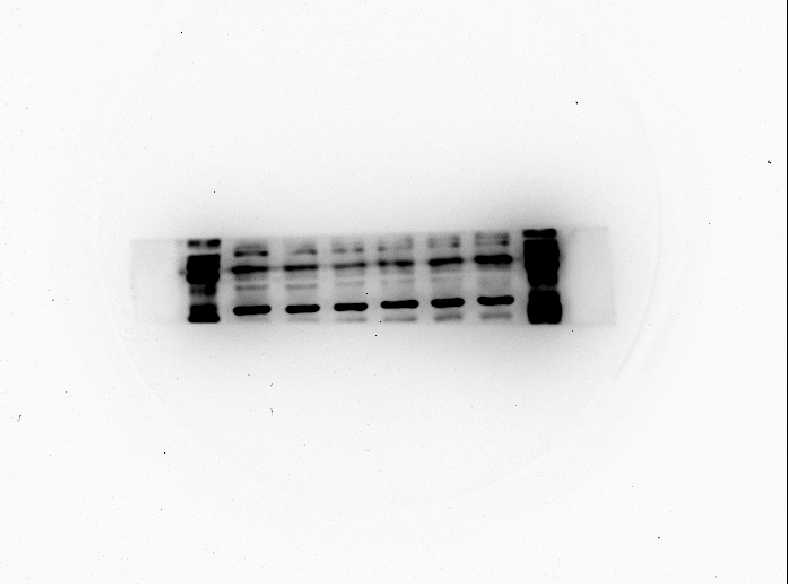

Supplement: Supplementary file 1 [file Data_Sheet_1.ZIP › the file of original Western Blot/figure6/p-Akt/Akt-2.jpg]

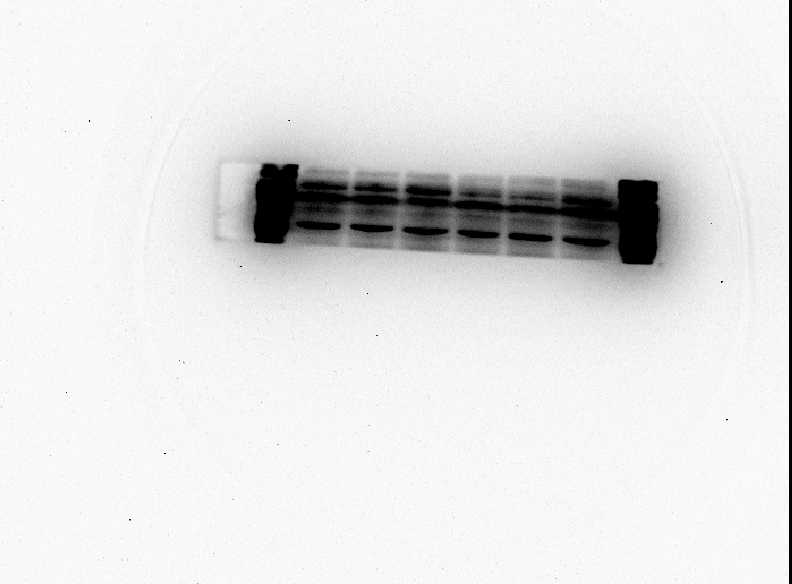

Supplement: Supplementary file 1 [file Data_Sheet_1.ZIP › the file of original Western Blot/figure6/p-Akt/Akt-3.jpg]

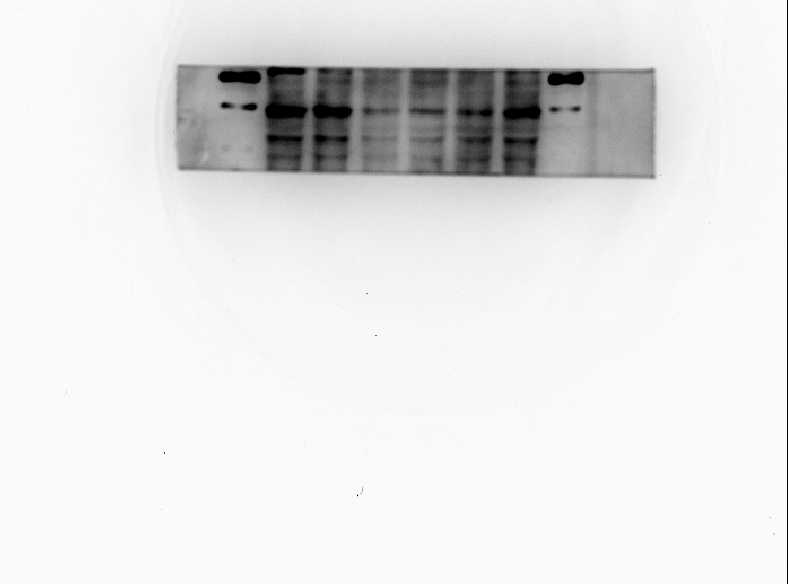

Supplement: Supplementary file 1 [file Data_Sheet_1.ZIP › the file of original Western Blot/figure6/p-Akt/p-Akt-1.jpg]

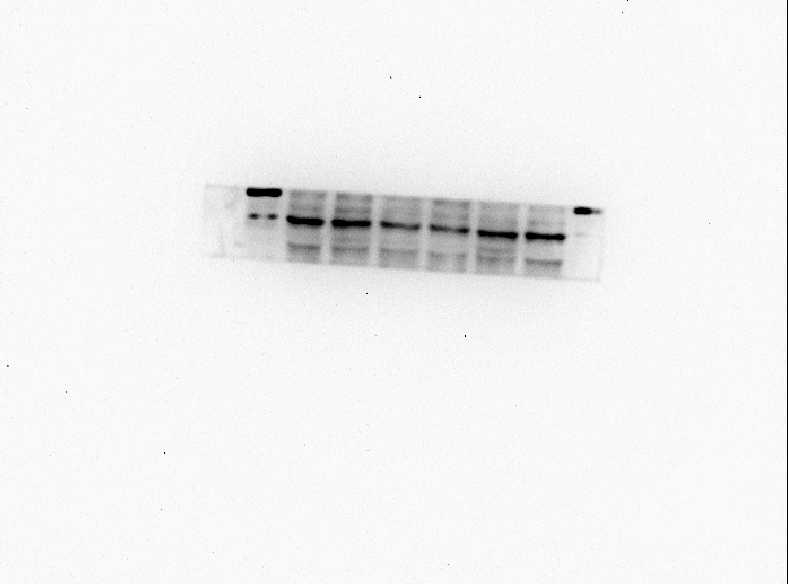

Supplement: Supplementary file 1 [file Data_Sheet_1.ZIP › the file of original Western Blot/figure6/p-Akt/p-Akt-2.jpg]

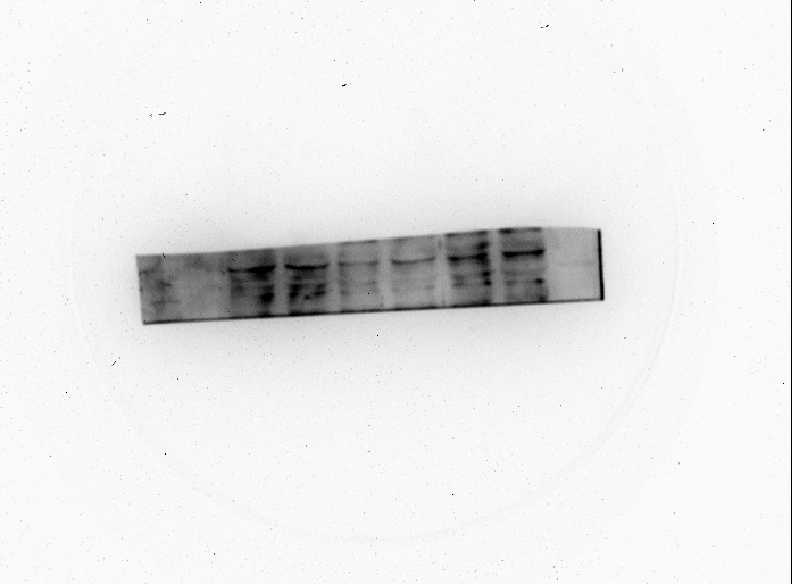

Supplement: Supplementary file 1 [file Data_Sheet_1.ZIP › the file of original Western Blot/figure6/p-Akt/p-Akt-3.jpg]

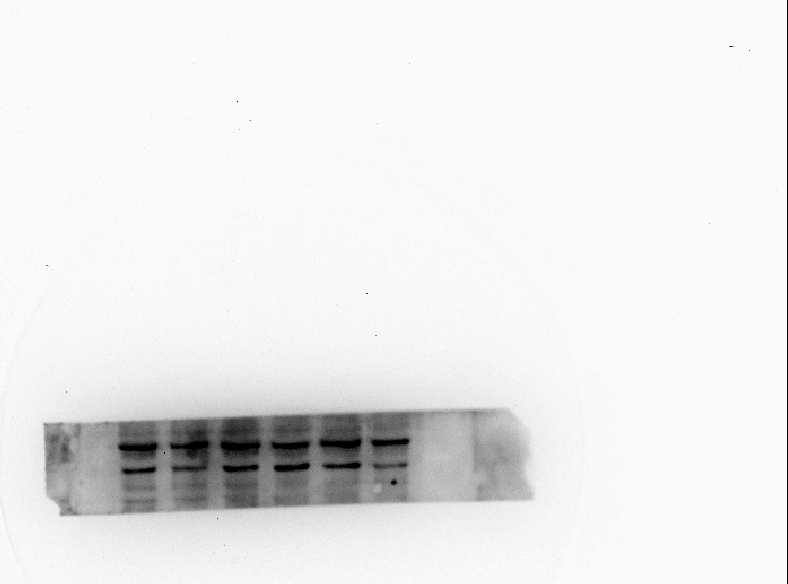

Supplement: Supplementary file 1 [file Data_Sheet_1.ZIP › the file of original Western Blot/figure6/p-CREB/CREB-1.jpg]

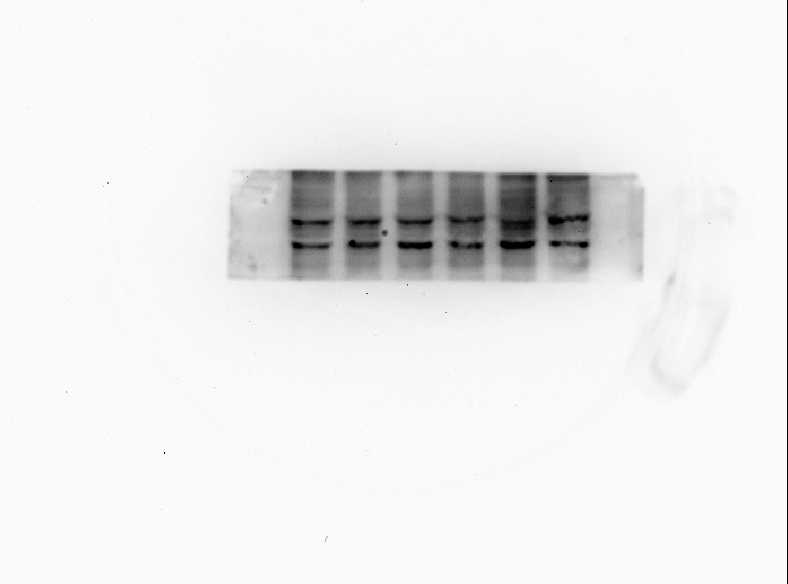

Supplement: Supplementary file 1 [file Data_Sheet_1.ZIP › the file of original Western Blot/figure6/p-CREB/CREB-2.jpg]

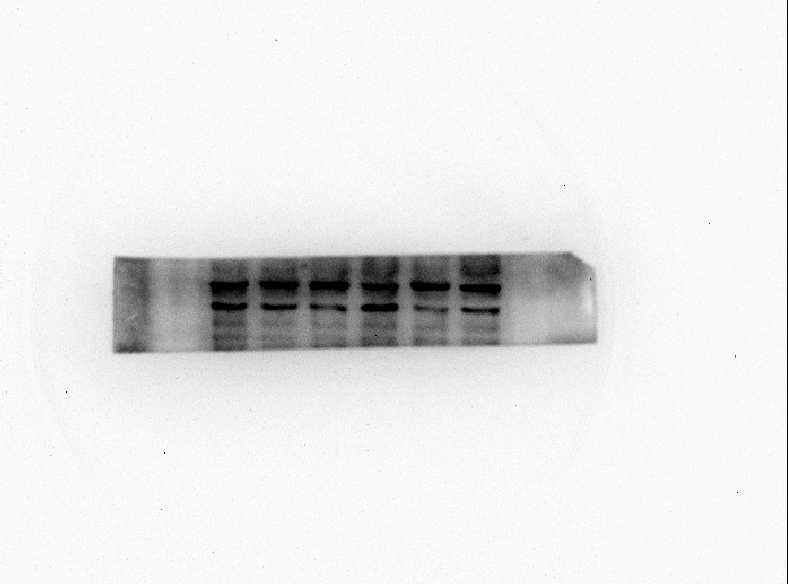

Supplement: Supplementary file 1 [file Data_Sheet_1.ZIP › the file of original Western Blot/figure6/p-CREB/CREB-3.jpg]

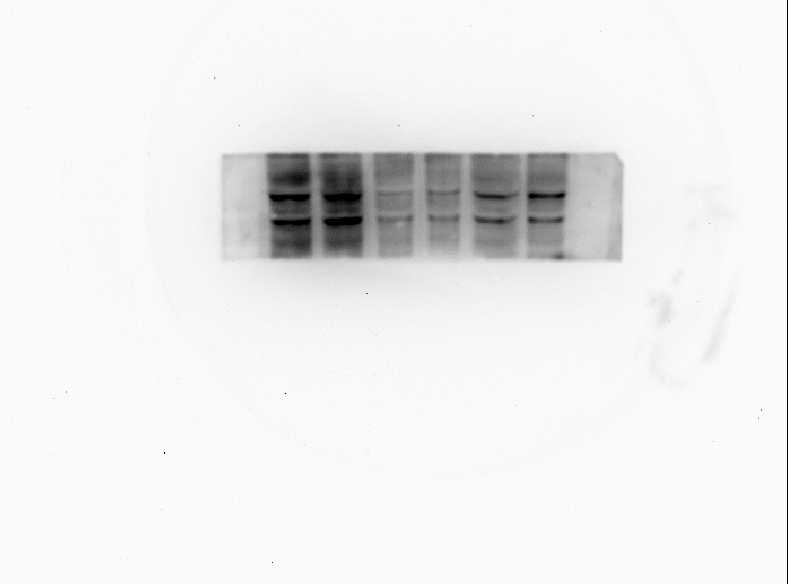

Supplement: Supplementary file 1 [file Data_Sheet_1.ZIP › the file of original Western Blot/figure6/p-CREB/p-CREB-1.jpg]

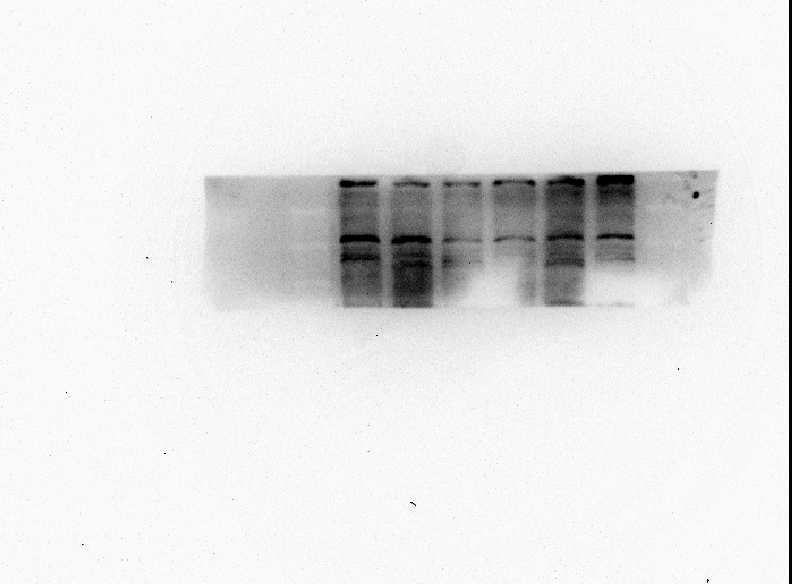

Supplement: Supplementary file 1 [file Data_Sheet_1.ZIP › the file of original Western Blot/figure6/p-CREB/p-CREB-2.jpg]

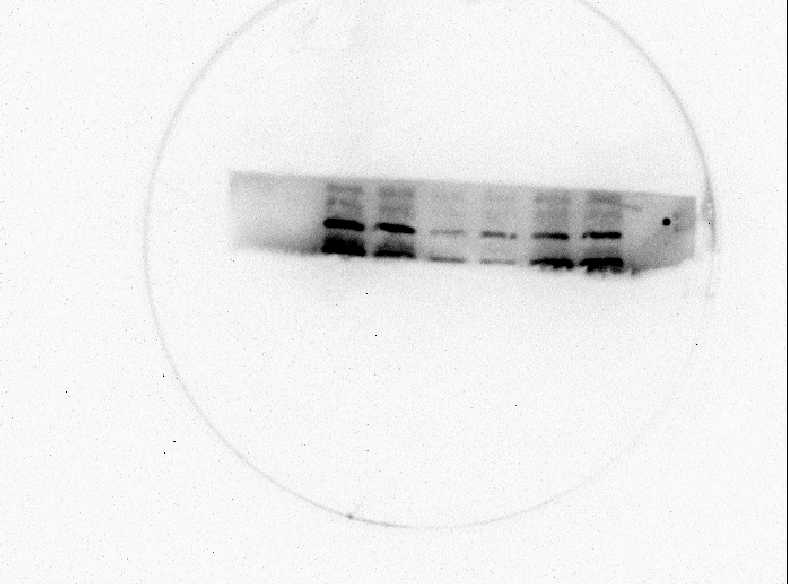

Supplement: Supplementary file 1 [file Data_Sheet_1.ZIP › the file of original Western Blot/figure6/p-CREB/p-CREB-3.jpg]

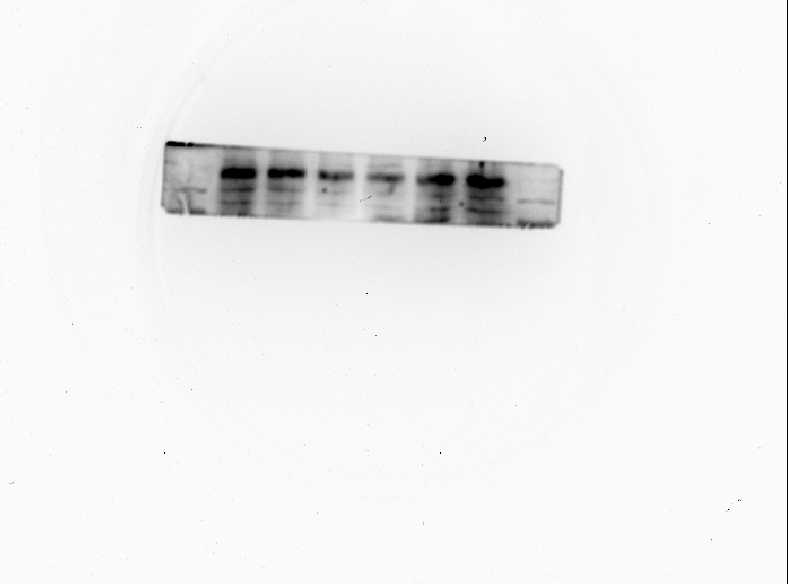

Supplement: Supplementary file 1 [file Data_Sheet_1.ZIP › the file of original Western Blot/figure6/TrkB/TrkB-1.jpg]

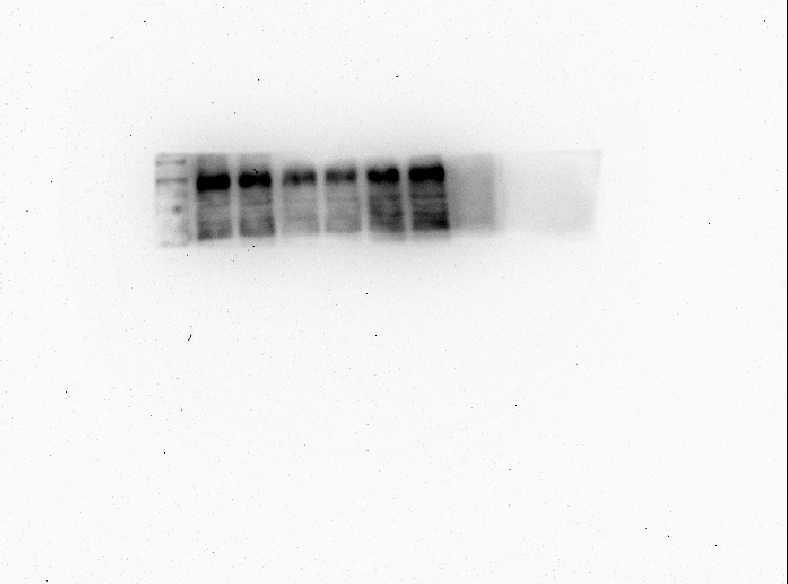

Supplement: Supplementary file 1 [file Data_Sheet_1.ZIP › the file of original Western Blot/figure6/TrkB/TrkB-2.jpg]

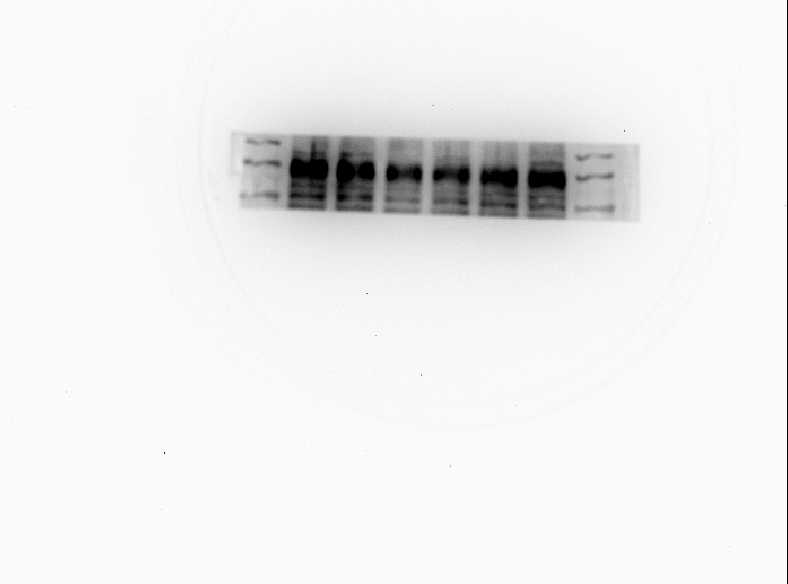

Supplement: Supplementary file 1 [file Data_Sheet_1.ZIP › the file of original Western Blot/figure6/TrkB/TrkB-3.jpg]

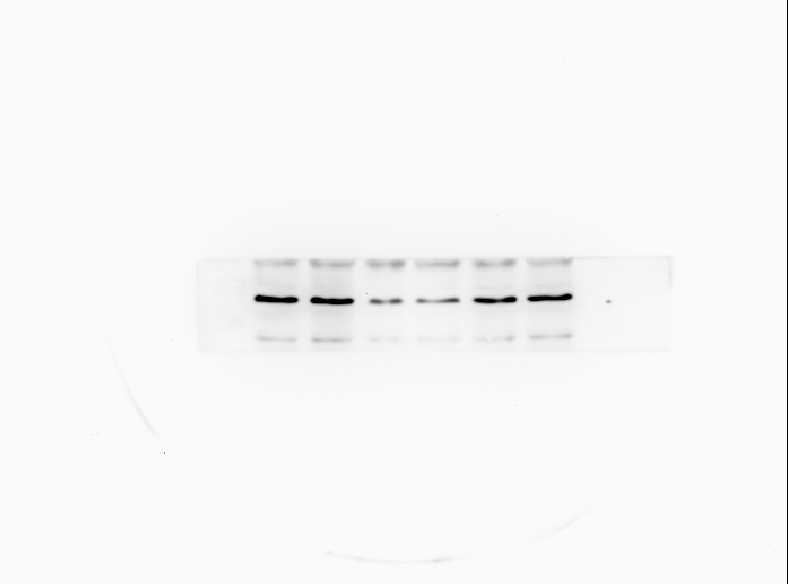

Supplement: Supplementary file 1 [file Data_Sheet_1.ZIP › the file of original Western Blot/figure7/PPARa┴/PPARa┴-1.jpg]

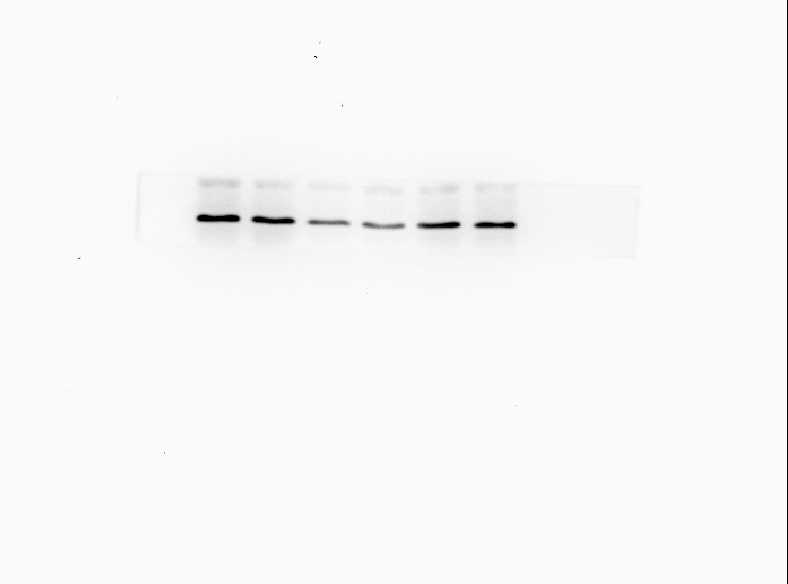

Supplement: Supplementary file 1 [file Data_Sheet_1.ZIP › the file of original Western Blot/figure7/PPARa┴/PPARa┴-2.jpg]

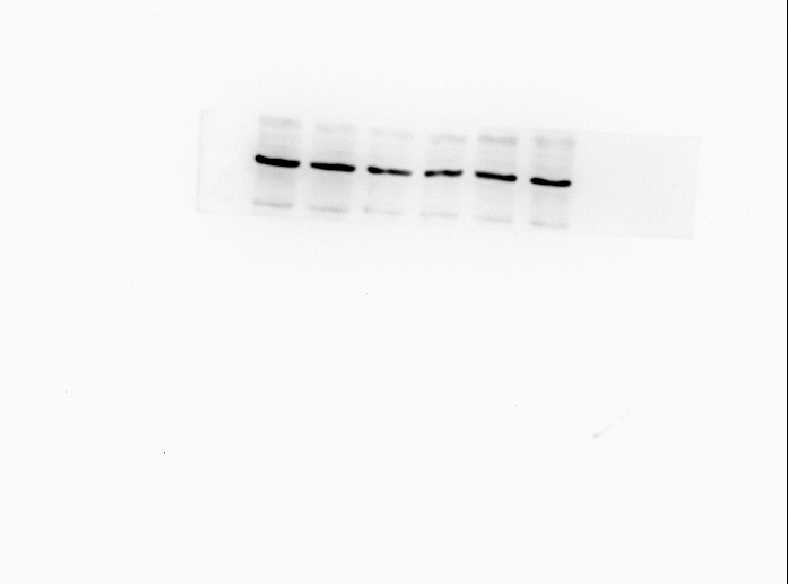

Supplement: Supplementary file 1 [file Data_Sheet_1.ZIP › the file of original Western Blot/figure7/PPARa┴/PPARa┴-3.jpg]

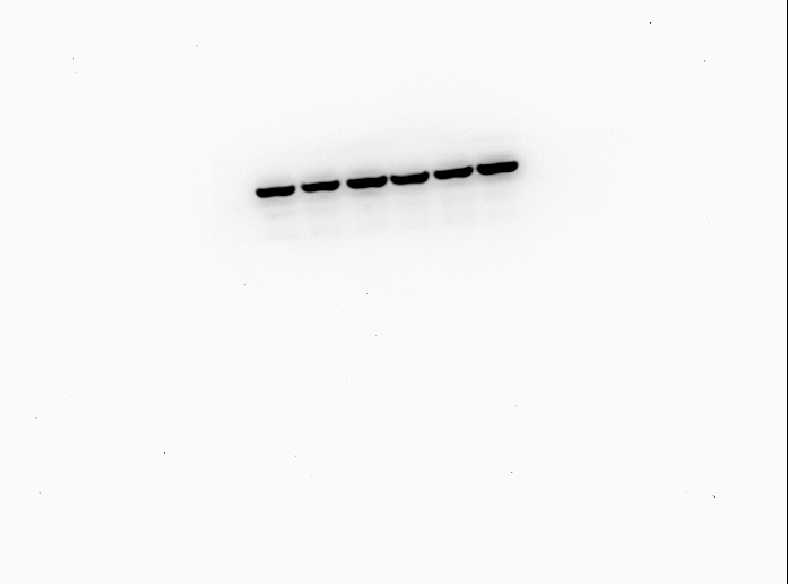

Supplement: Supplementary file 1 [file Data_Sheet_1.ZIP › the file of original Western Blot/figure7/PPARa┴/a┬-actin-1.jpg]

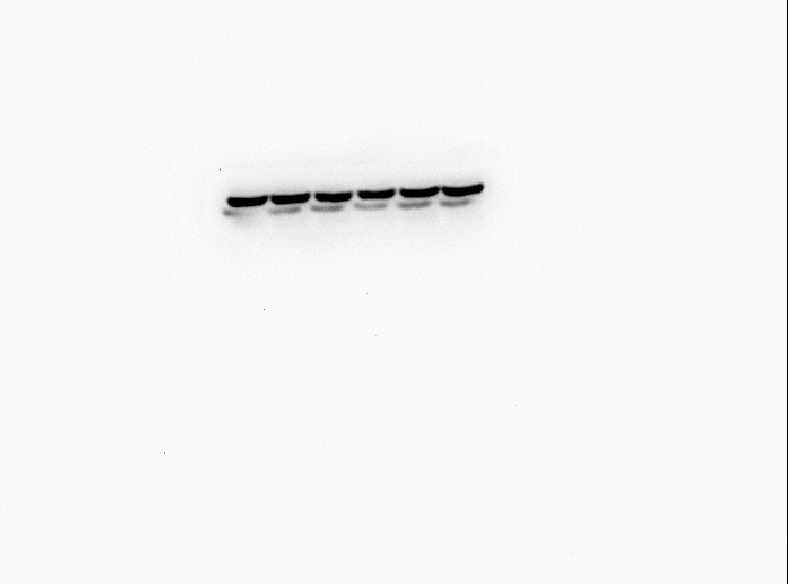

Supplement: Supplementary file 1 [file Data_Sheet_1.ZIP › the file of original Western Blot/figure7/PPARa┴/a┬-actin-2.jpg]

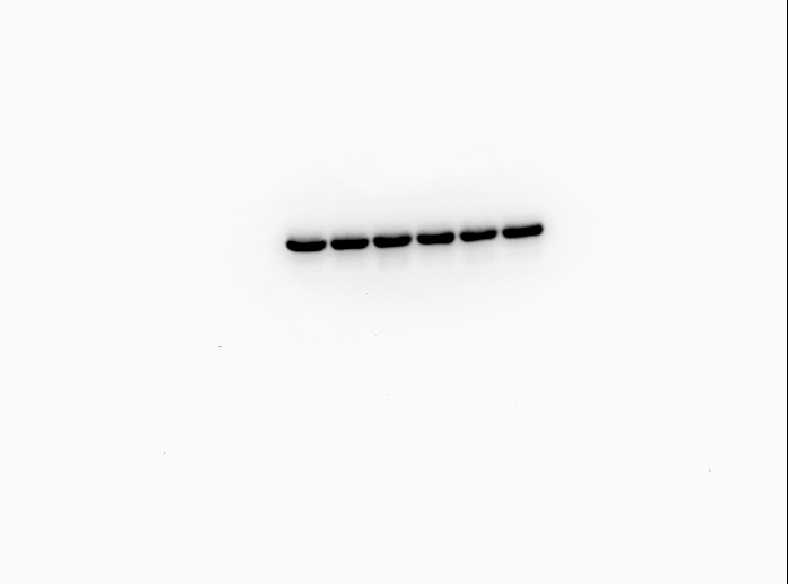

Supplement: Supplementary file 1 [file Data_Sheet_1.ZIP › the file of original Western Blot/figure7/PPARa┴/a┬-actin-3.jpg]

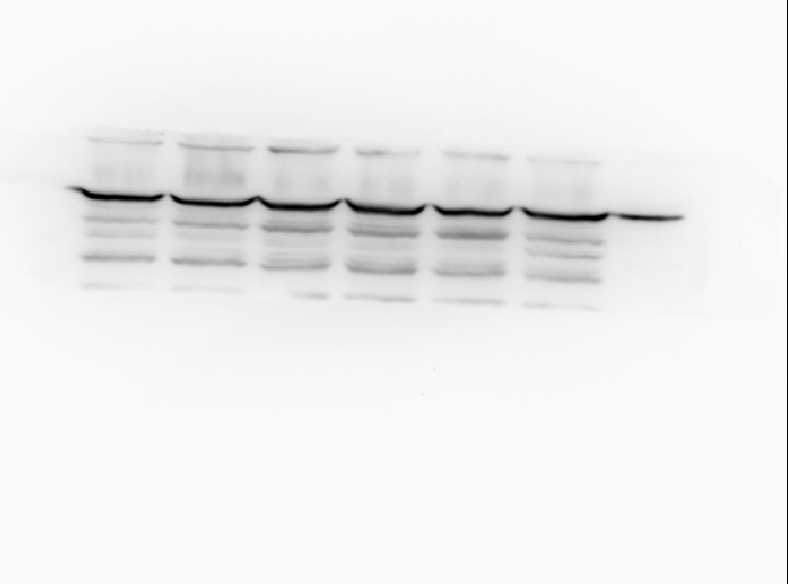

Supplement: Supplementary file 1 [file Data_Sheet_1.ZIP › the file of original Western Blot/figure7/PPARa┬/PPARa┬-1.jpg]

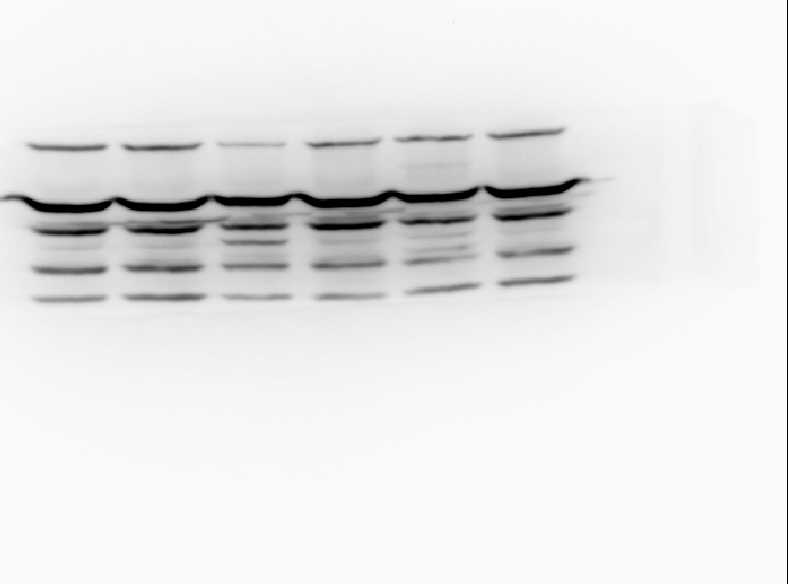

Supplement: Supplementary file 1 [file Data_Sheet_1.ZIP › the file of original Western Blot/figure7/PPARa┬/PPARa┬-2.jpg]

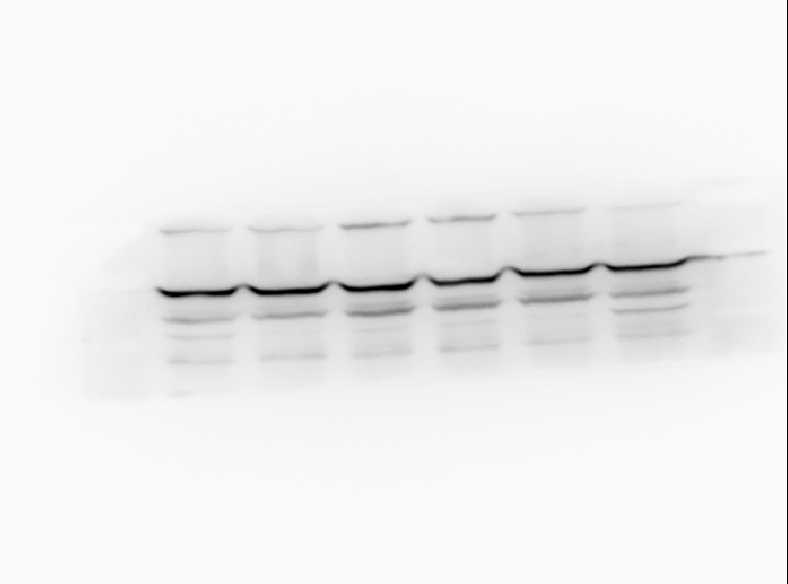

Supplement: Supplementary file 1 [file Data_Sheet_1.ZIP › the file of original Western Blot/figure7/PPARa┬/PPARa┬-3.jpg]

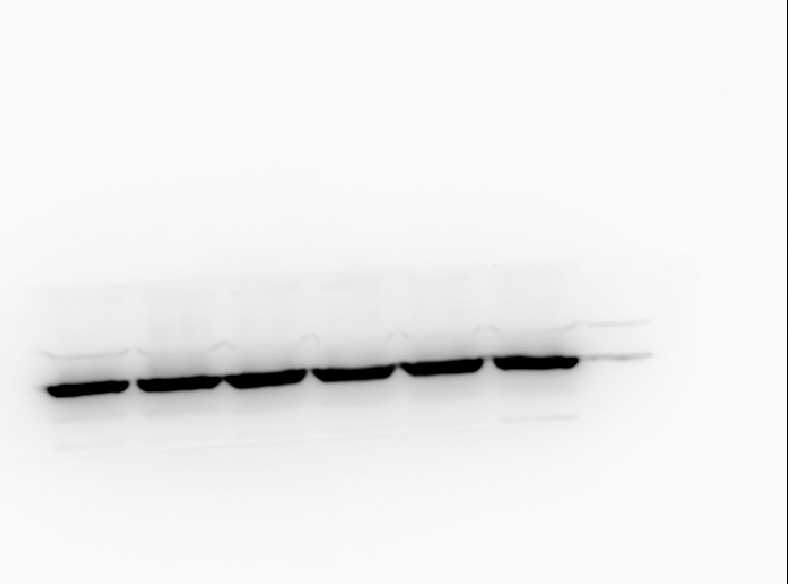

Supplement: Supplementary file 1 [file Data_Sheet_1.ZIP › the file of original Western Blot/figure7/PPARa┬/a┬-actin-1.jpg]

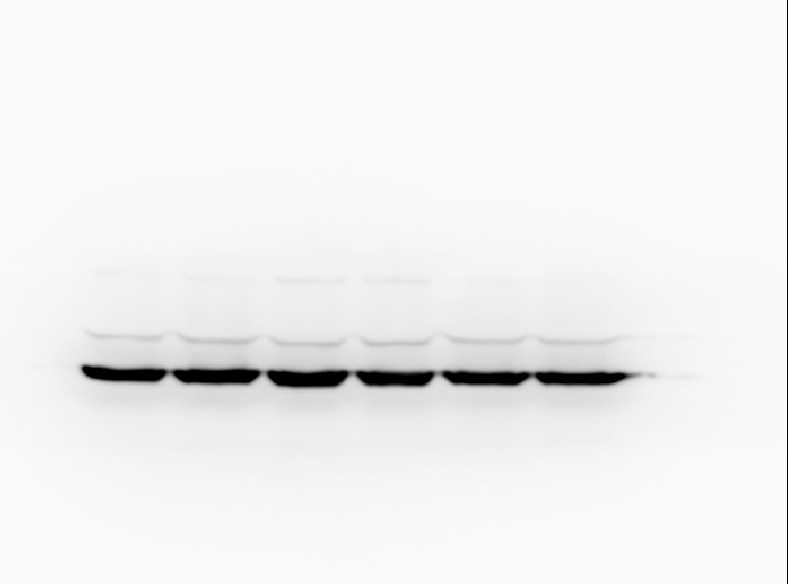

Supplement: Supplementary file 1 [file Data_Sheet_1.ZIP › the file of original Western Blot/figure7/PPARa┬/a┬-actin-2.jpg]

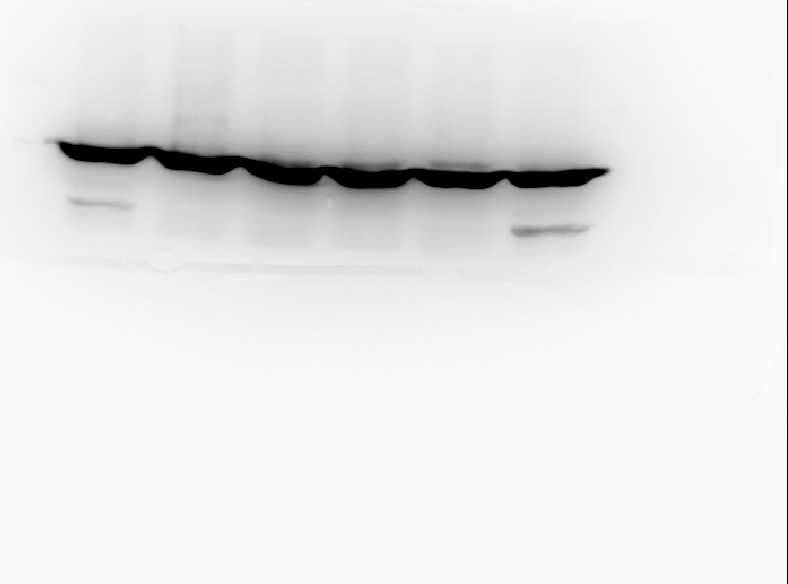

Supplement: Supplementary file 1 [file Data_Sheet_1.ZIP › the file of original Western Blot/figure7/PPARa┬/a┬-actin-3.jpg]

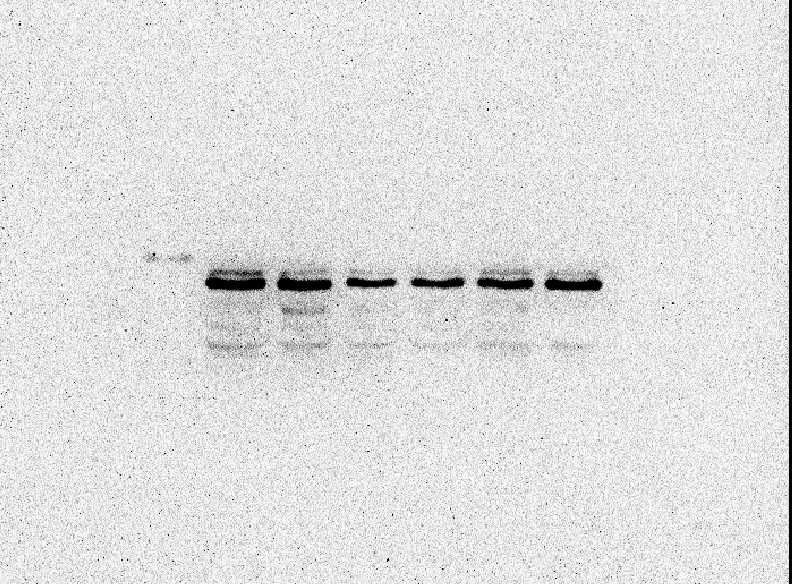

Supplement: Supplementary file 1 [file Data_Sheet_1.ZIP › the file of original Western Blot/figure7/PPARa├/PPARa├-1.jpg]

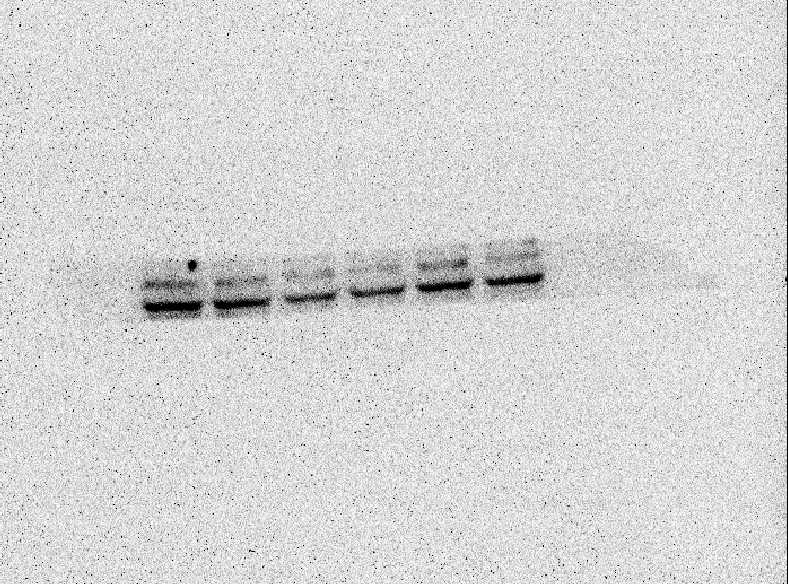

Supplement: Supplementary file 1 [file Data_Sheet_1.ZIP › the file of original Western Blot/figure7/PPARa├/PPARa├-2.jpg]

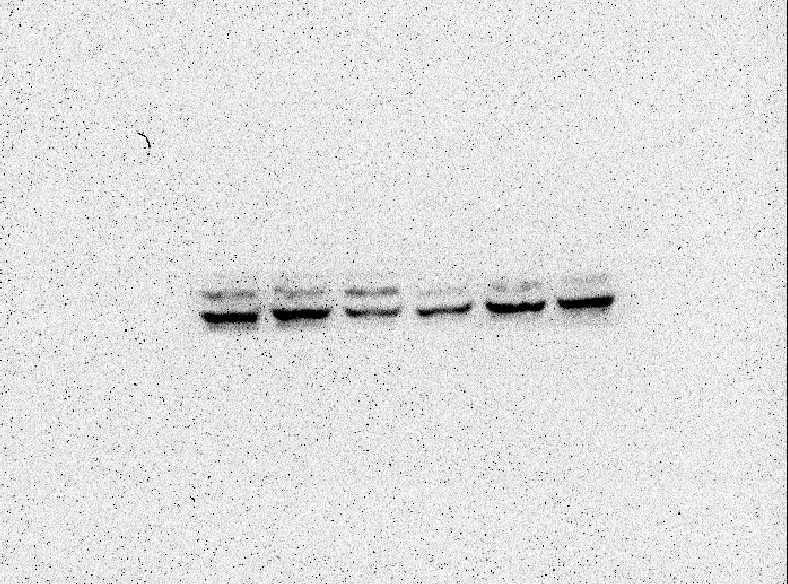

Supplement: Supplementary file 1 [file Data_Sheet_1.ZIP › the file of original Western Blot/figure7/PPARa├/PPARa├-3.jpg]

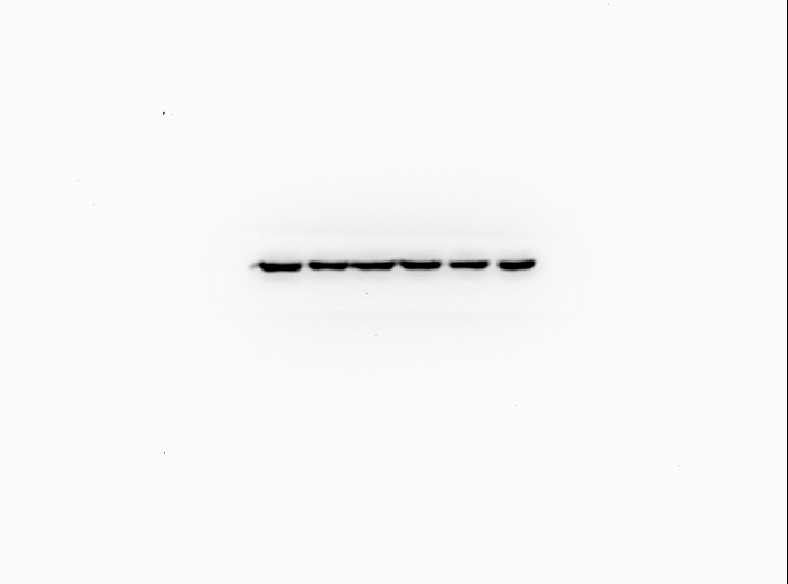

Supplement: Supplementary file 1 [file Data_Sheet_1.ZIP › the file of original Western Blot/figure7/PPARa├/a┬-actin-1.jpg]

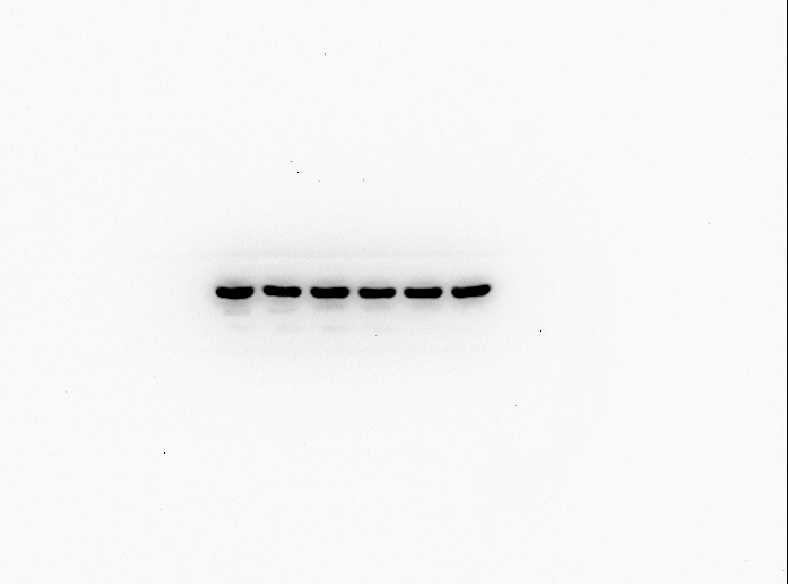

Supplement: Supplementary file 1 [file Data_Sheet_1.ZIP › the file of original Western Blot/figure7/PPARa├/a┬-actin-2.jpg]

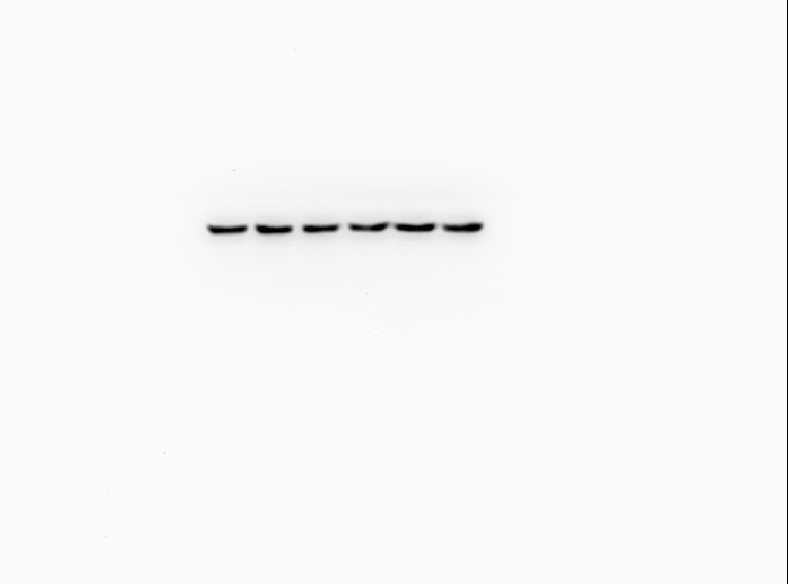

Supplement: Supplementary file 1 [file Data_Sheet_1.ZIP › the file of original Western Blot/figure7/PPARa├/a┬-actin-3.jpg]

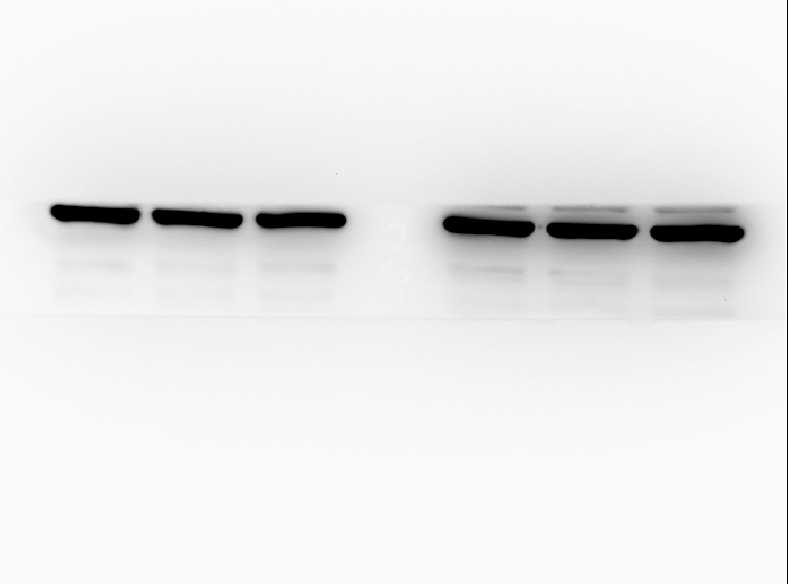

Supplement: Supplementary file 1 [file Data_Sheet_1.ZIP › the file of original Western Blot/figure8/PPARa┴/GAPDH-1 and GAPDH-2.jpg]

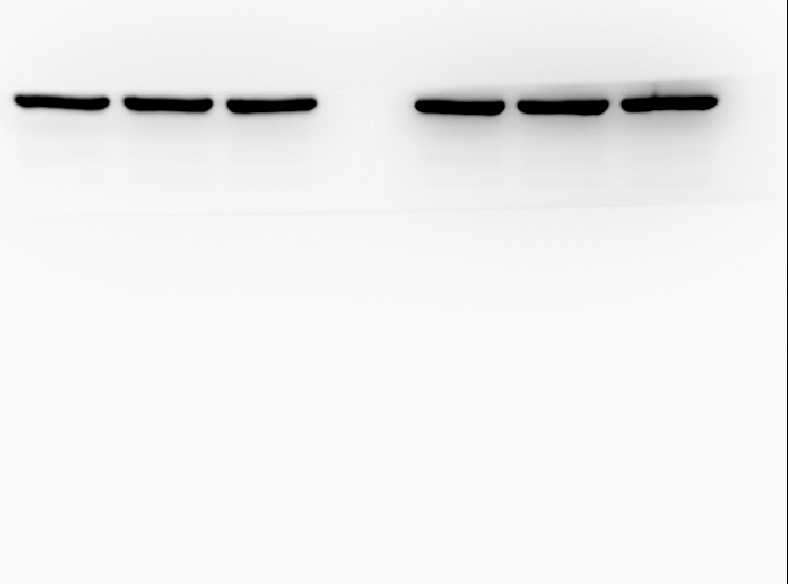

Supplement: Supplementary file 1 [file Data_Sheet_1.ZIP › the file of original Western Blot/figure8/PPARa┴/GAPDH-3 and GAPDH-4.jpg]

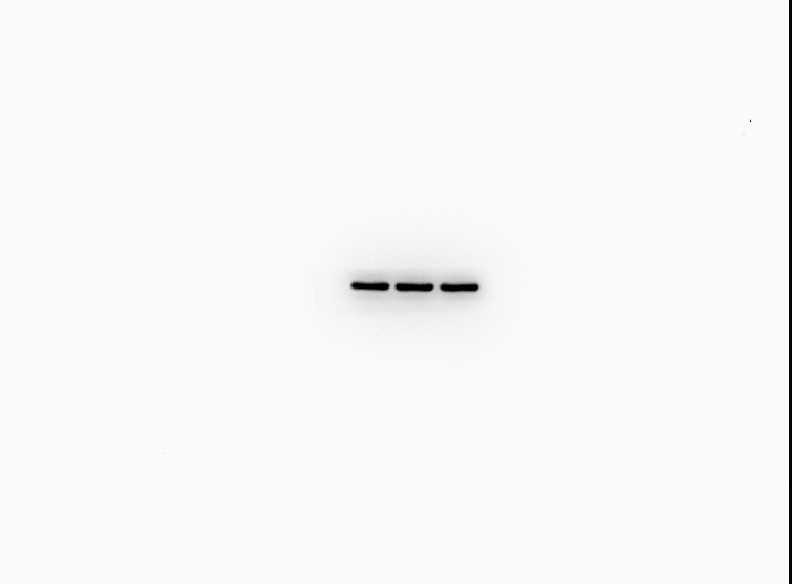

Supplement: Supplementary file 1 [file Data_Sheet_1.ZIP › the file of original Western Blot/figure8/PPARa┴/GAPDH-5.jpg]

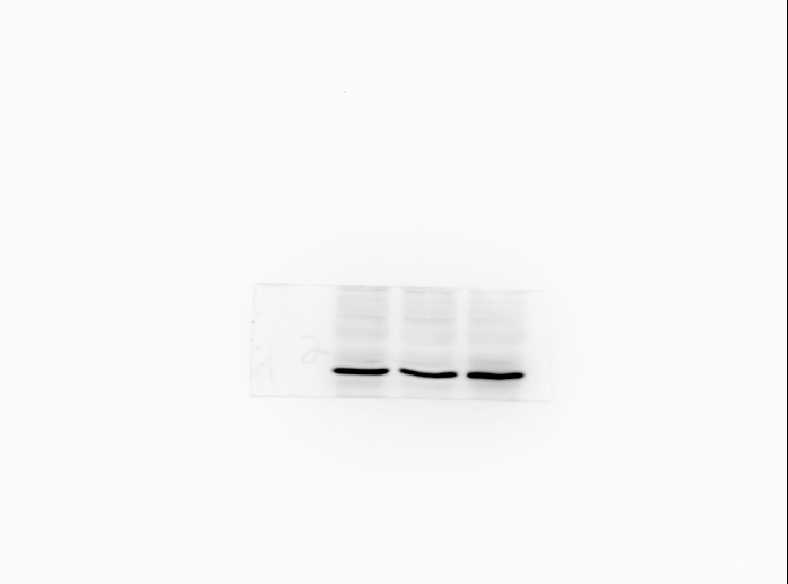

Supplement: Supplementary file 1 [file Data_Sheet_1.ZIP › the file of original Western Blot/figure8/PPARa┴/PPARa┴-1.jpg]

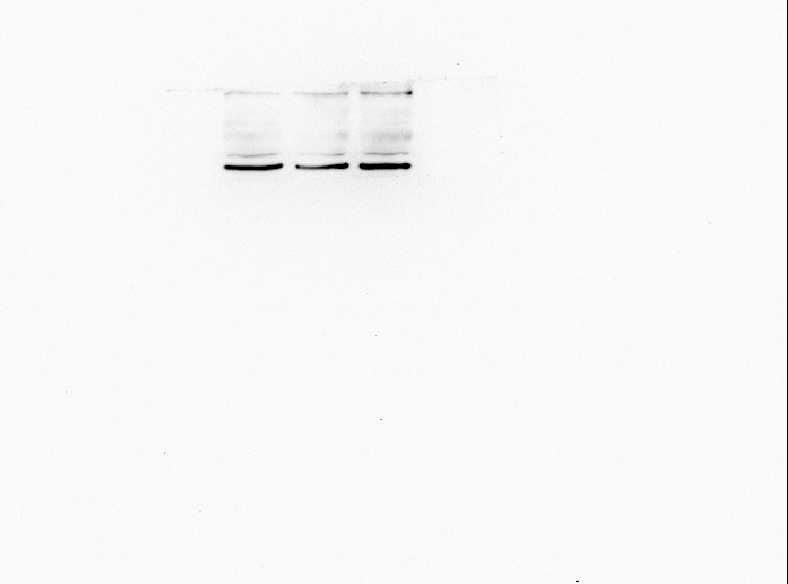

Supplement: Supplementary file 1 [file Data_Sheet_1.ZIP › the file of original Western Blot/figure8/PPARa┴/PPARa┴-2.jpg]

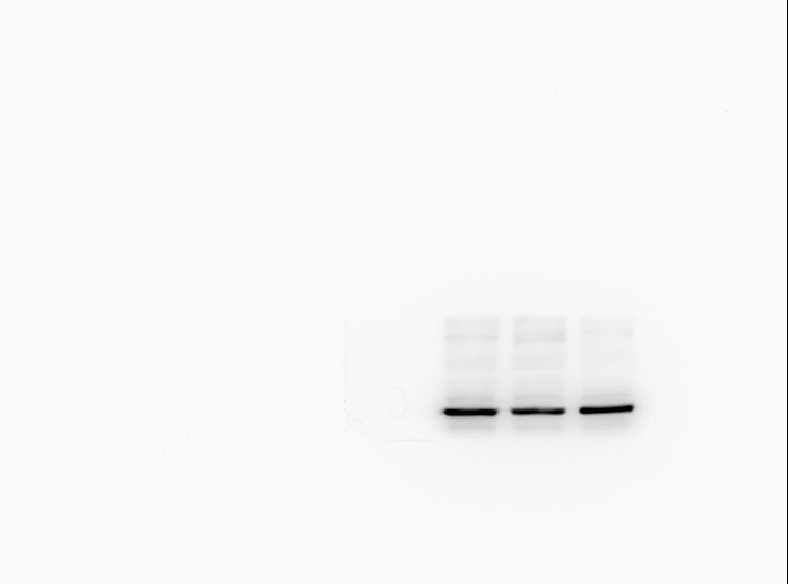

Supplement: Supplementary file 1 [file Data_Sheet_1.ZIP › the file of original Western Blot/figure8/PPARa┴/PPARa┴-3.jpg]

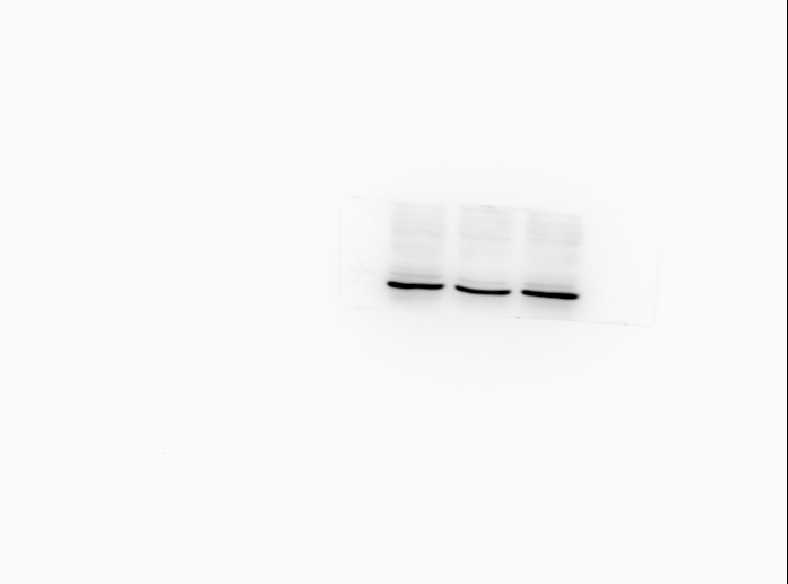

Supplement: Supplementary file 1 [file Data_Sheet_1.ZIP › the file of original Western Blot/figure8/PPARa┴/PPARa┴-4.jpg]

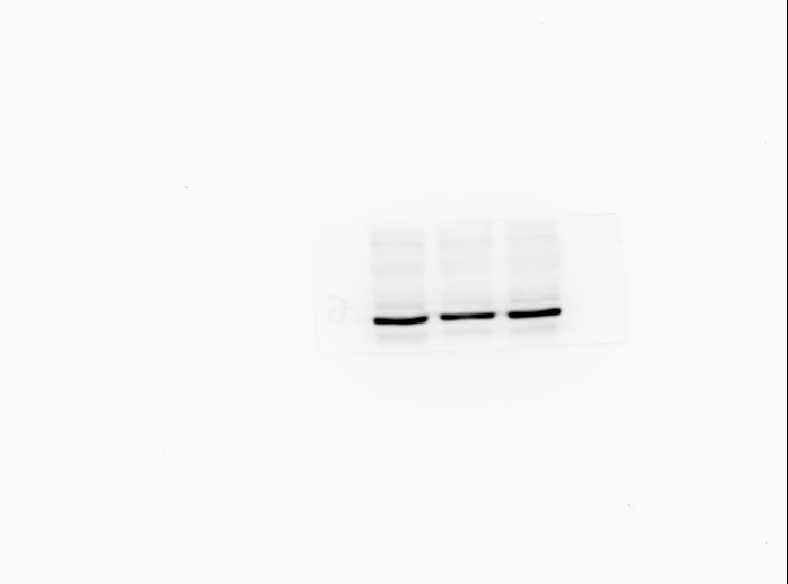

Supplement: Supplementary file 1 [file Data_Sheet_1.ZIP › the file of original Western Blot/figure8/PPARa┴/PPARa┴-5.jpg]

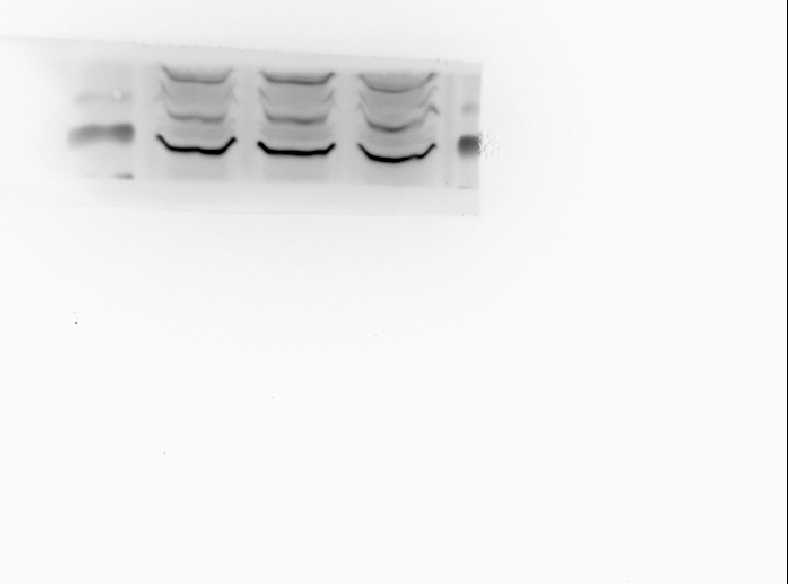

Supplement: Supplementary file 1 [file Data_Sheet_1.ZIP › the file of original Western Blot/figure8/PPARa┬/PPARa┬-1.jpg]

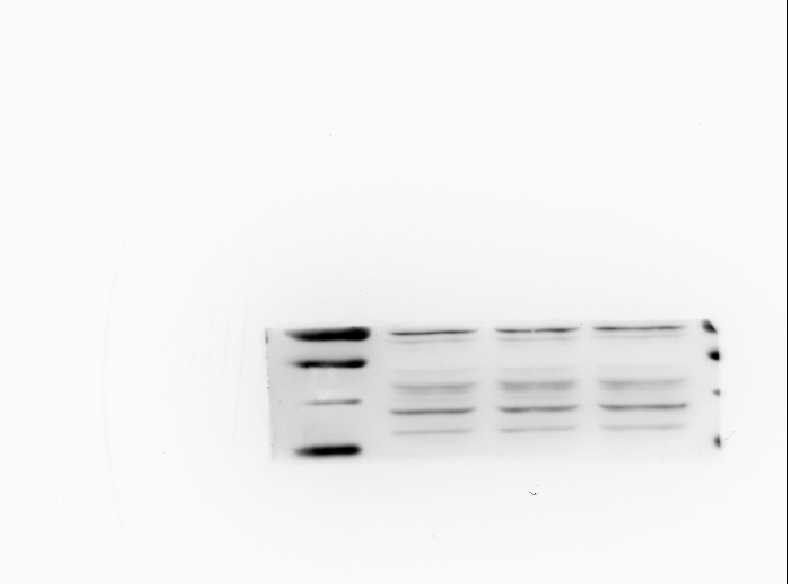

Supplement: Supplementary file 1 [file Data_Sheet_1.ZIP › the file of original Western Blot/figure8/PPARa┬/PPARa┬-2.jpg]

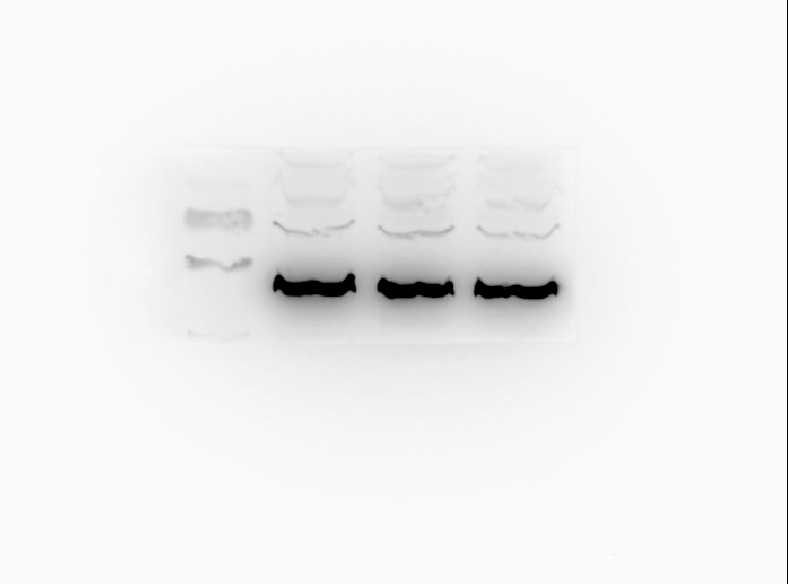

Supplement: Supplementary file 1 [file Data_Sheet_1.ZIP › the file of original Western Blot/figure8/PPARa┬/PPARa┬-3.jpg]

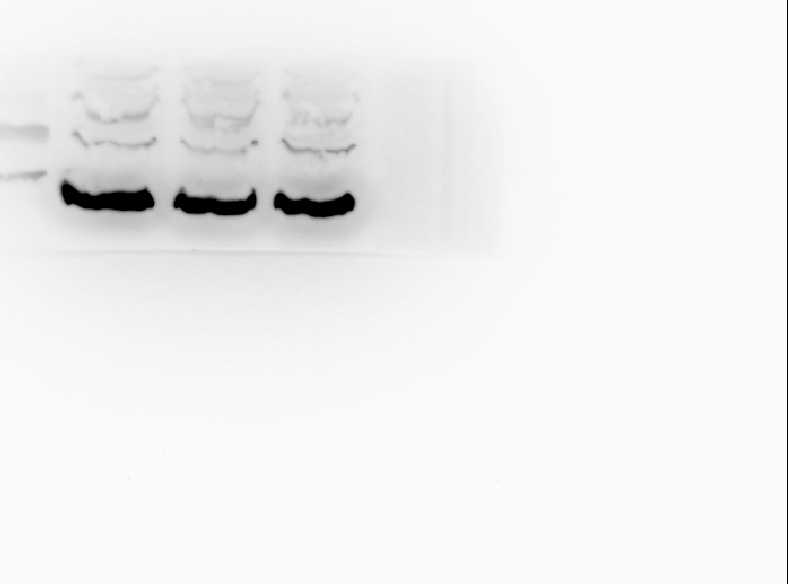

Supplement: Supplementary file 1 [file Data_Sheet_1.ZIP › the file of original Western Blot/figure8/PPARa┬/PPARa┬-4.jpg]

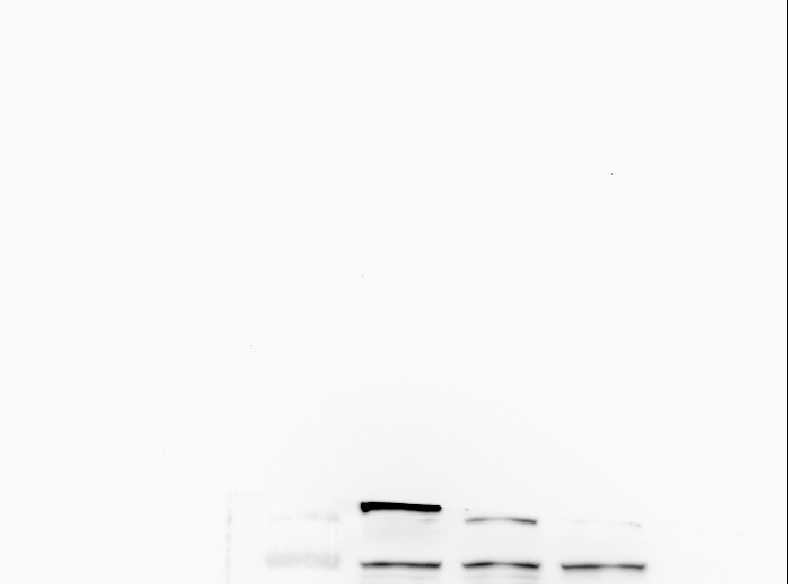

Supplement: Supplementary file 1 [file Data_Sheet_1.ZIP › the file of original Western Blot/figure8/PPARa┬/PPARa┬-5.jpg]

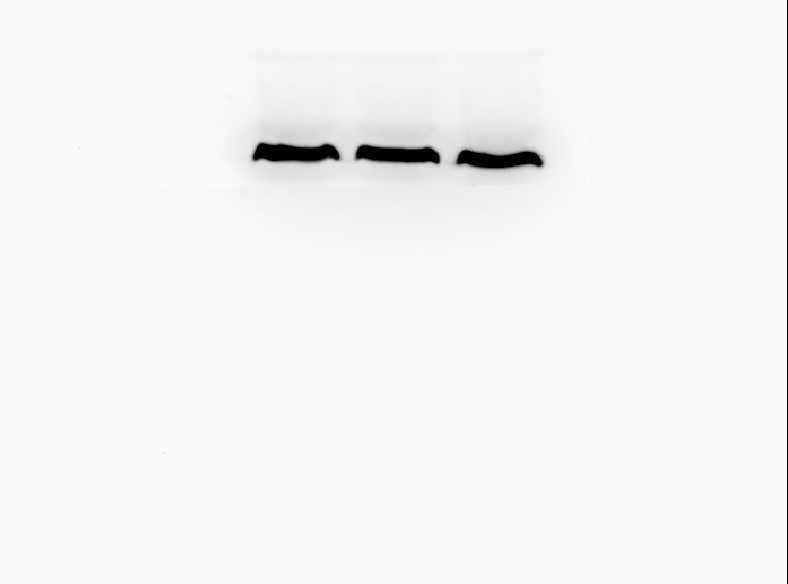

Supplement: Supplementary file 1 [file Data_Sheet_1.ZIP › the file of original Western Blot/figure8/PPARa┬/a┬-actin-1.jpg]

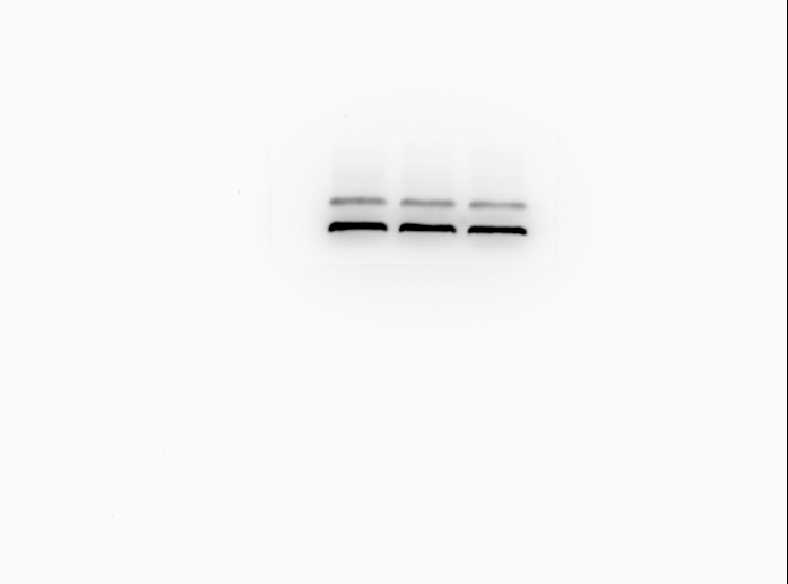

Supplement: Supplementary file 1 [file Data_Sheet_1.ZIP › the file of original Western Blot/figure8/PPARa┬/a┬-actin-2.jpg]

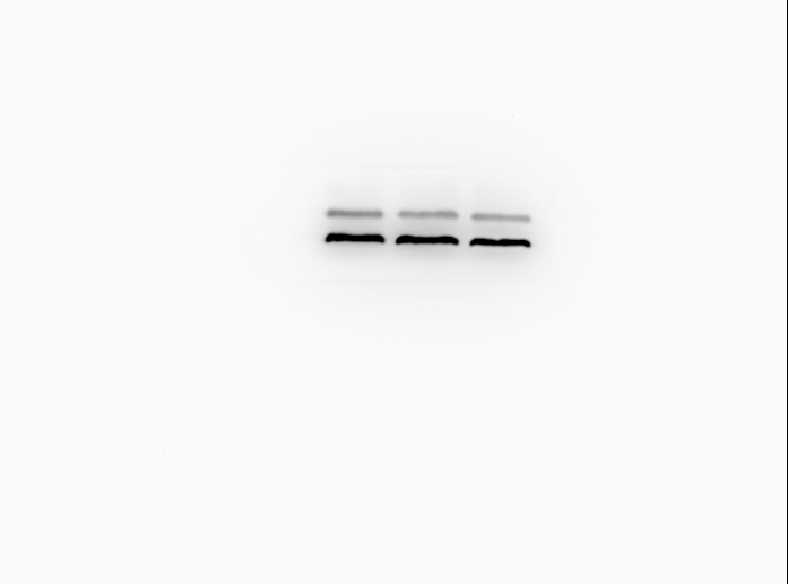

Supplement: Supplementary file 1 [file Data_Sheet_1.ZIP › the file of original Western Blot/figure8/PPARa┬/a┬-actin-3.jpg]

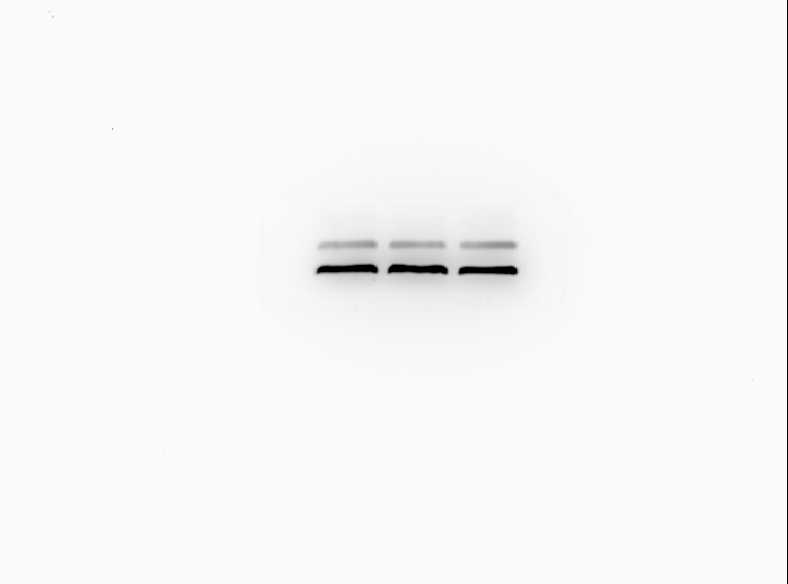

Supplement: Supplementary file 1 [file Data_Sheet_1.ZIP › the file of original Western Blot/figure8/PPARa┬/a┬-actin-4.jpg]

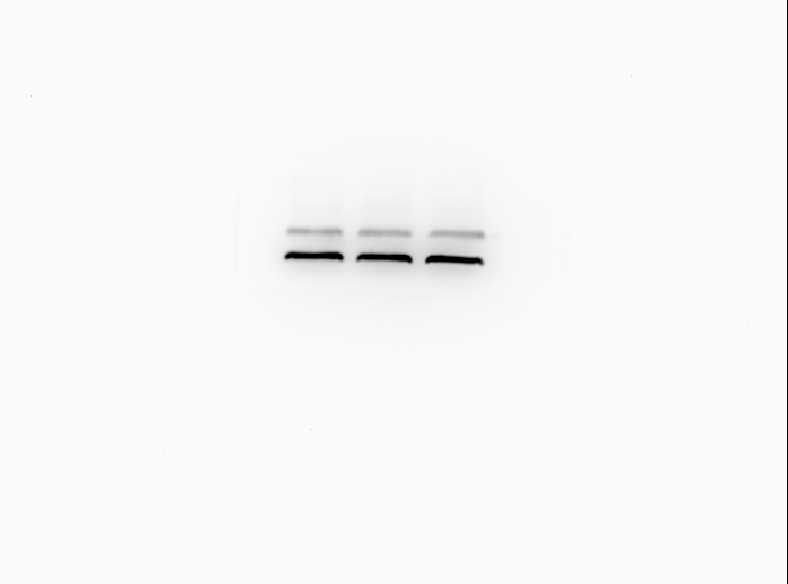

Supplement: Supplementary file 1 [file Data_Sheet_1.ZIP › the file of original Western Blot/figure8/PPARa┬/a┬-actin-5.jpg]

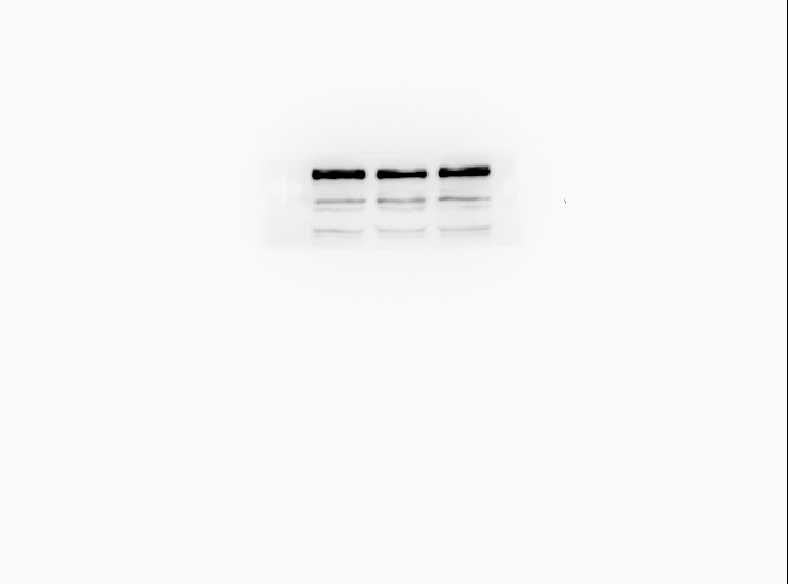

Supplement: Supplementary file 1 [file Data_Sheet_1.ZIP › the file of original Western Blot/figure8/PPARa├/PPARa├-1.jpg]

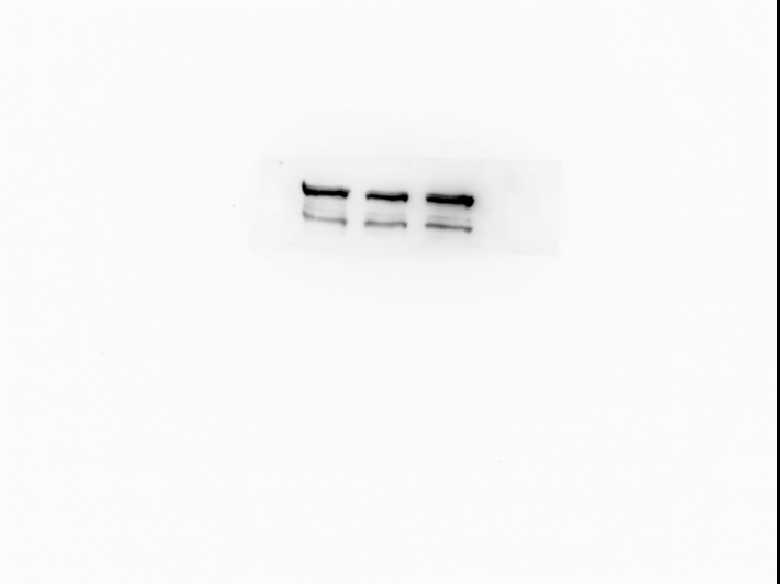

Supplement: Supplementary file 1 [file Data_Sheet_1.ZIP › the file of original Western Blot/figure8/PPARa├/PPARa├-2.jpg]

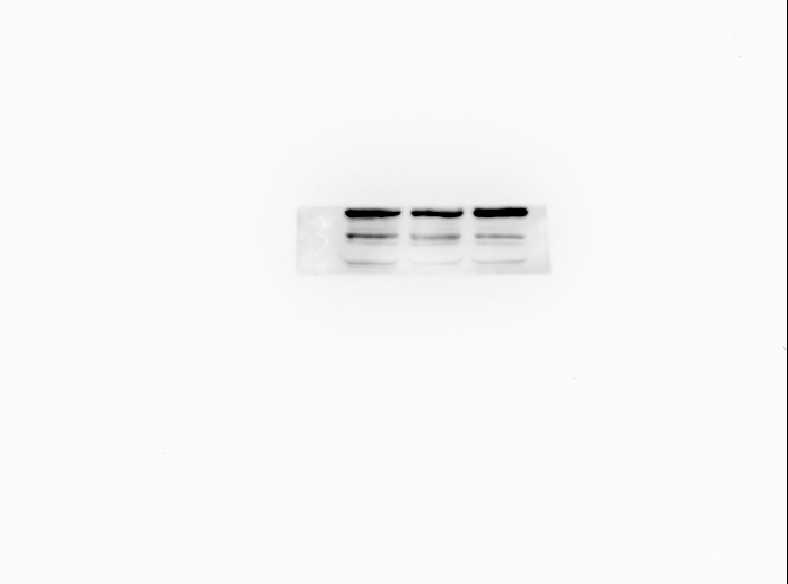

Supplement: Supplementary file 1 [file Data_Sheet_1.ZIP › the file of original Western Blot/figure8/PPARa├/PPARa├-3.jpg]

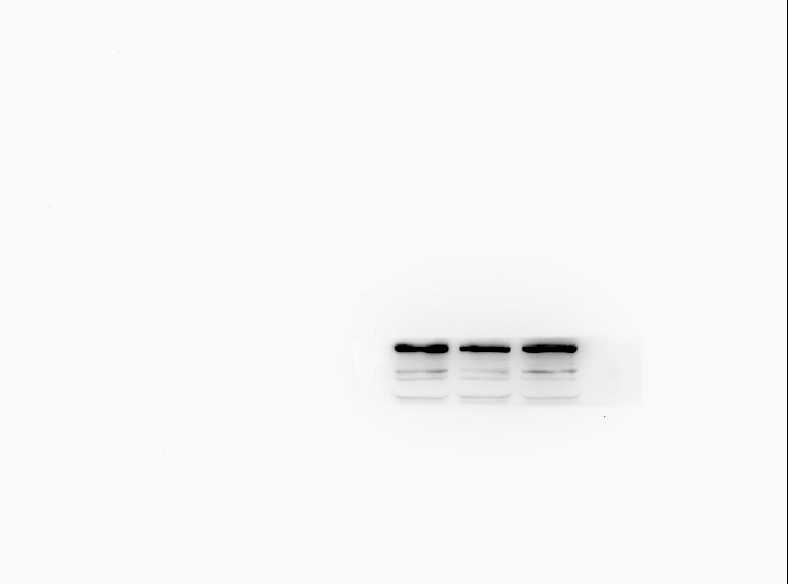

Supplement: Supplementary file 1 [file Data_Sheet_1.ZIP › the file of original Western Blot/figure8/PPARa├/PPARa├-4.jpg]

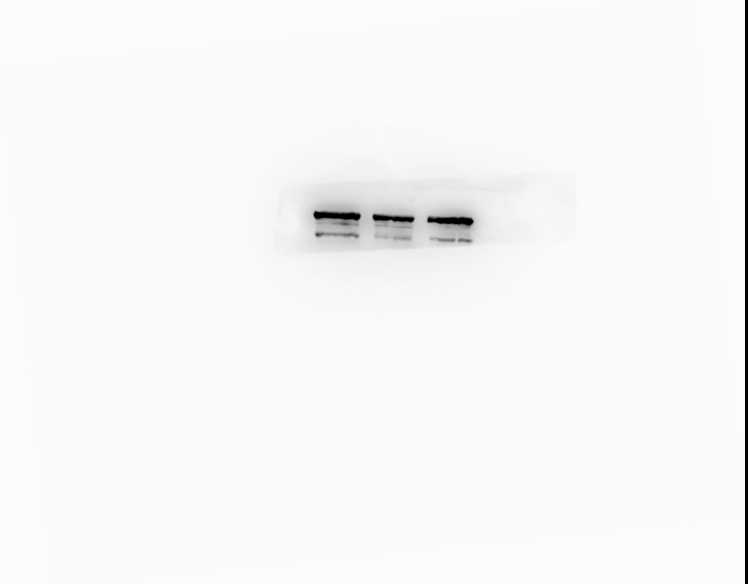

Supplement: Supplementary file 1 [file Data_Sheet_1.ZIP › the file of original Western Blot/figure8/PPARa├/PPARa├-5.jpg]

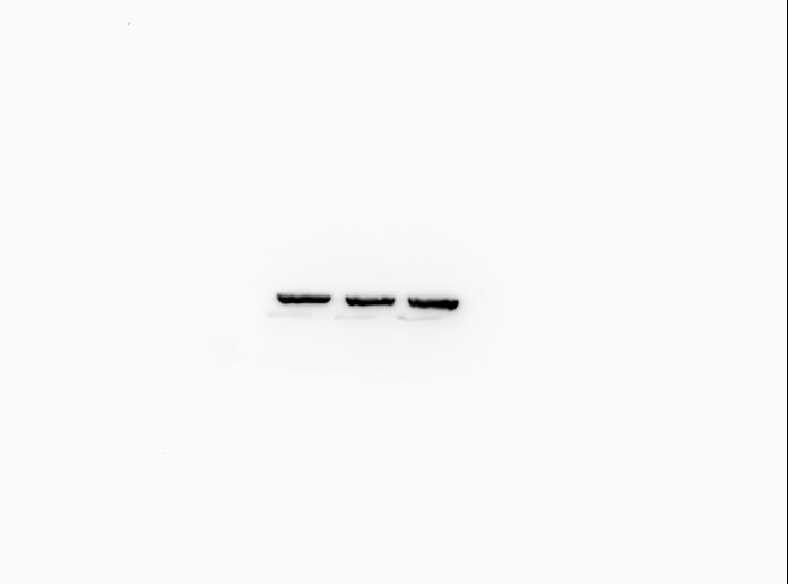

Supplement: Supplementary file 1 [file Data_Sheet_1.ZIP › the file of original Western Blot/figure8/PPARa├/a┬-actin-1.jpg]

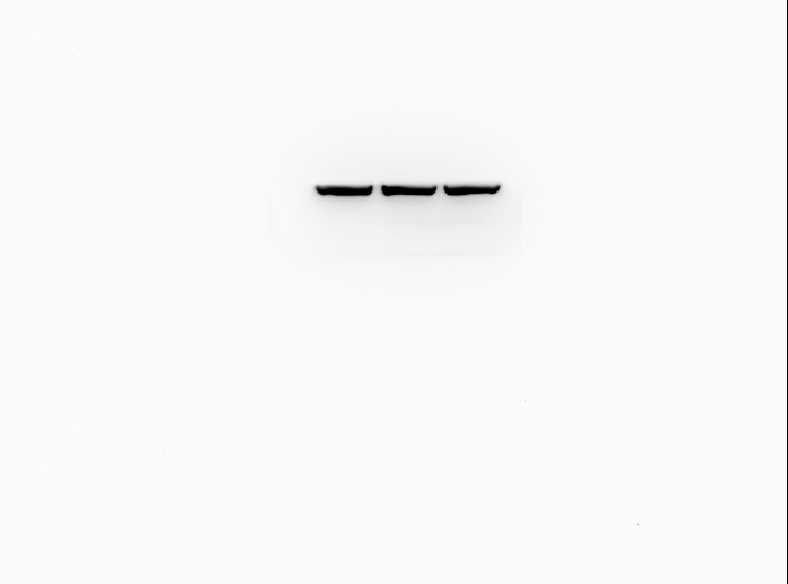

Supplement: Supplementary file 1 [file Data_Sheet_1.ZIP › the file of original Western Blot/figure8/PPARa├/a┬-actin-2.jpg]

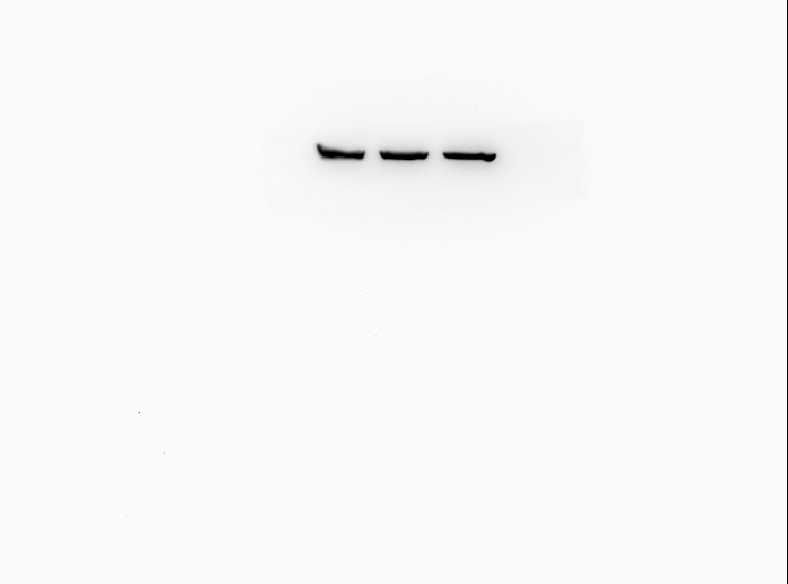

Supplement: Supplementary file 1 [file Data_Sheet_1.ZIP › the file of original Western Blot/figure8/PPARa├/a┬-actin-3.jpg]

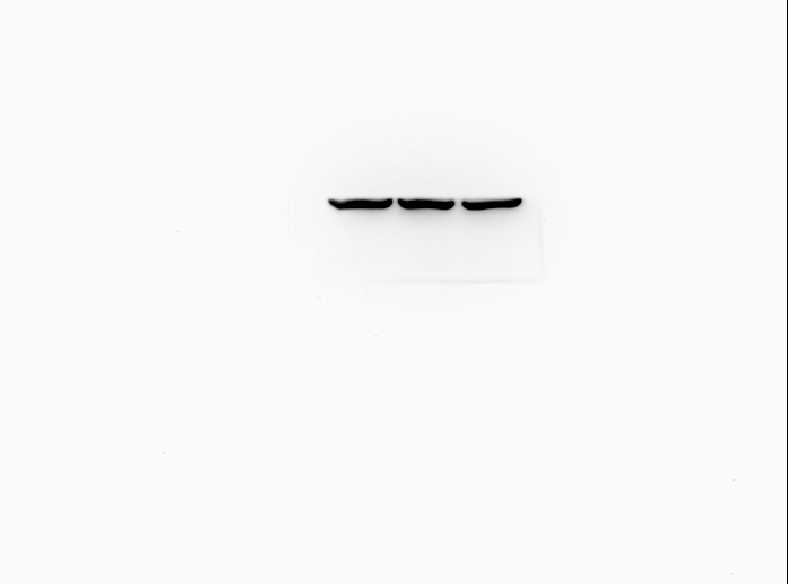

Supplement: Supplementary file 1 [file Data_Sheet_1.ZIP › the file of original Western Blot/figure8/PPARa├/a┬-actin-4.jpg]

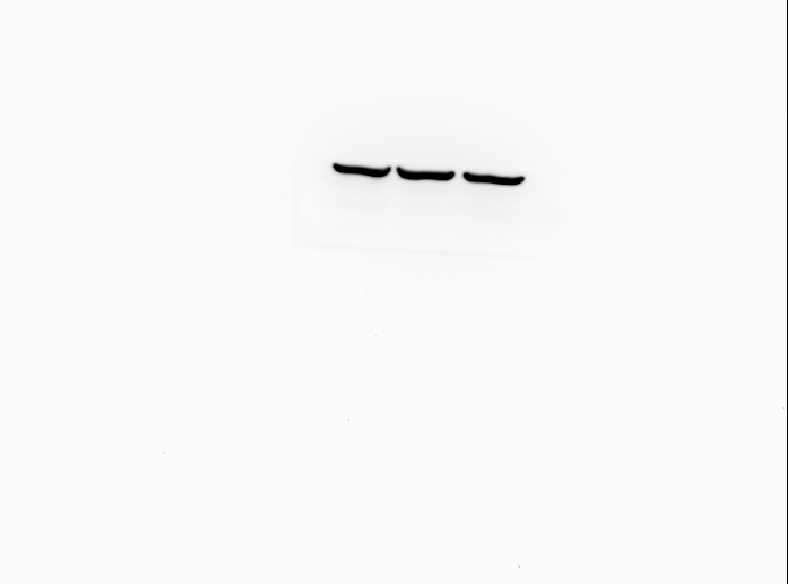

Supplement: Supplementary file 1 [file Data_Sheet_1.ZIP › the file of original Western Blot/figure8/PPARa├/a┬-actin-5.jpg]
